# modCHIMERA: a novel murine closed-head model of moderate traumatic brain injury

**DOI:** 10.1038/s41598-018-25737-6

**Published:** 2018-05-16

**Authors:** A. D. Sauerbeck, C. Fanizzi, J. H. Kim, M. Gangolli, P. V. Bayly, C. L. Wellington, D. L. Brody, T. T. Kummer

**Affiliations:** 1 Department of Neurology, Washington University School of Medicine in St. Louis, Missouri St. Louis, USA; 2 Department of Neurosurgery, Fondazione IRCCS Ca’ Granda Ospedale Maggiore Policlinico, Milan, Italy; 3 Department of Biomedical Engineering, Washington University School of Medicine in St. Louis, Missouri St. Louis, USA; 4 Mechanical Engineering and Materials Science, Washington University in St. Louis, Missouri St. Louis, USA; 5 Department of Pathology and Laboratory Medicine, University of British Columbia, Vancouver, Canada

## Abstract

Traumatic brain injury is a major source of global disability and mortality. Preclinical TBI models are a crucial component of therapeutic investigation. We report a tunable, monitored model of murine non-surgical, diffuse closed-head injury—modCHIMERA—characterized by impact as well as linear and rotational acceleration. modCHIMERA is based on the Closed-Head Impact Model of Engineered Rotational Acceleration (CHIMERA) platform. We tested this model at 2 energy levels: 1.7 and 2.1 Joules—substantially higher than previously reported for this system. Kinematic analysis demonstrated linear acceleration exceeding injury thresholds in humans, although outcome metrics tracked impact energy more closely than kinematic parameters. Acute severity metrics were consistent with a complicated-mild or moderate TBI, a clinical population characterized by high morbidity but potentially reversible pathology. Axonal injury was multifocal and bilateral, neuronal death was detected in the hippocampus, and microglial neuroinflammation was prominent. Acute functional analysis revealed prolonged post-injury unconsciousness, and decreased spontaneous behavior and stimulated neurological scores. Neurobehavioral deficits were demonstrated in spatial learning/memory and socialization at 1-month. The overall injury profile of modCHIMERA corresponds with the range responsible for a substantial portion of TBI-related disability in humans. modCHIMERA should provide a reliable platform for efficient analysis of TBI pathophysiology and testing of treatment modalities.

## Introduction

Traumatic brain injury (TBI) occurs in 2.4 million people per year in the United States^1^ and has been described by the Centers for Disease Control and Prevention as a ‘silent epidemic’ with an annual cost of $76.5 billion in 2010^2,3^. Globally TBI is the leading cause of death and disability for adolescents and younger adults, and its incidence is rising^4^. Despite the tremendous public health impact of brain trauma, TBI research has yet to produce an effective therapy. As a result, despite shifting demographics of injury, no clear improvement in overall outcomes including mortality has occurred in the past two decades^5^.

Approximately 75% of TBI is considered “mild” or concussive^6^. Criteria defining mild TBI have been published by several international organizations^6–10^. While not perfectly aligned, these definitions stipulate that patients with mild TBI have a Glasgow coma scale (GCS^11^) of 13–15 and may also experience a short period of unconsciousness (in most definitions under 30 minutes) and/or post-traumatic amnesia (generally defined as less than 24 hours), and sometimes additional transient neurological dysfunction. Recognizing the broad spectrum of injury that can meet these definitions, a subclass of more significantly-injured mild TBI patients is often identified by the presence of one or more “complications.” Such complicated-mild TBI patients are generally identified by the presence of a radiologically detectable brain injury^12–14^, but have also been defined as exhibiting a GCS at the lower end of the mild spectrum^15^, prolonged loss of consciousness or post-traumatic amnesia, and/or electroencephalographic evidence of brain injury^16^.

The great majority of mild TBI patients make an excellent recovery. Those patients at the complicated-mild end of the spectrum often do significantly worse, with longer return to work times^17^ and poorer performance on neuropsychological tests even to 23 years post-injury^16,18^. More than 30% of complicated-mild TBI patients suffer moderate to severe disability by Glasgow Outcome Scale and 82% present with symptoms on the Rivermead Postconcussion Symptoms Questionnaire^13^. Indeed, outcomes after complicated-mild TBI are reported to be more similar to those after moderate than mild TBI^12^. Thus patients with complicated-mild TBI bear a disproportionate burden of the impairment attributed to TBI classically defined as mild.

This burden is shared with moderate and severe TBI patients, groups that are often considered together though they encompass a tremendous range of severities and heterogeneity of injuries. Moderate TBI in particular is poorly-understood and under-studied^19^. Similar to mild TBI, other than a widely-accepted GCS range of 9–12, moderate TBI definitions vary^6–10^. Loss of consciousness may range from 30 minutes to 24 hours and post-traumatic amnesia may last as long as 7 days. Resultantly, prevalence and outcomes of moderate TBI are challenging to determine, though it has been estimated that perhaps 20% of TBI in the United States is moderate (4–28% depending on study)^19^. Patients with moderate TBI have an overall mortality of 15%, and only 20% recover without significant disability^19^. Some have suggested that moderate TBI patients suffer from many of the same pathophysiological processes that characterize severe TBI, but that this subgroup may be more amenable to intervention making them an attractive focus for therapeutic development^20^.

Although widely understood as, at best, approximations of human brain trauma, experimental animal models will continue to play an important role in understanding TBI and in testing potential treatment modalities. Many animal models of TBI exist^21–23^—each involves trade-offs. While large animal models better replicate human physiology and share a gyrencephalic brain, their carrying costs and long generational time are prominent hindrances. Rodent models are therefore more widely utilized. Mouse models in particular offer the potential to make use of an extremely diverse transgenic tool set for *in vivo* observation and genetic dissection of injury pathways. To maximize face validity preclinical models should ideally include both impact- and inertial-elements (including both linear and rotational acceleration) during TBI, as both are generally present in human brain trauma and may interact. Effective models should also produce a reliable injury, be accomplished quickly, minimize or eliminate surgical procedures due to the inherent effect such procedures and their mandatory anesthesia protocols have on brain physiology, and respect ethical requirements for the treatment of experimental animals.

We report a non-surgical murine model of diffuse, closed-head injury called modCHIMERA. modCHIMERA is based on the CHIMERA platform, previously utilized as a model of mild, repetitive TBI^24^. modCHIMERA was designed to expand the range of injury severities achievable with this platform, by targeting the complicated-mild/moderate span of the TBI severity spectrum that is responsible for a substantial portion of potentially-reversible TBI-related disability in humans. modCHIMERA is characterized by direct impact followed by semi-restrained linear and rotational acceleration. modCHIMERA furthermore incorporates additional key strengths of the CHIMERA platform, namely a highly-reliable, tunable, and monitored injury mechanism of defined energy. We describe the modifications required to develop modCHIMERA, including the implementation of skull and spine protection, and its kinematic properties. We show that modCHIMERA results in multi-domain neuropathological and functional alterations consistent with a complicated-mild/moderate TBI, and thus may serve as a useful model of these debilitating but likely intervenable injuries.

## Results

### Model development

**Figure 1:**
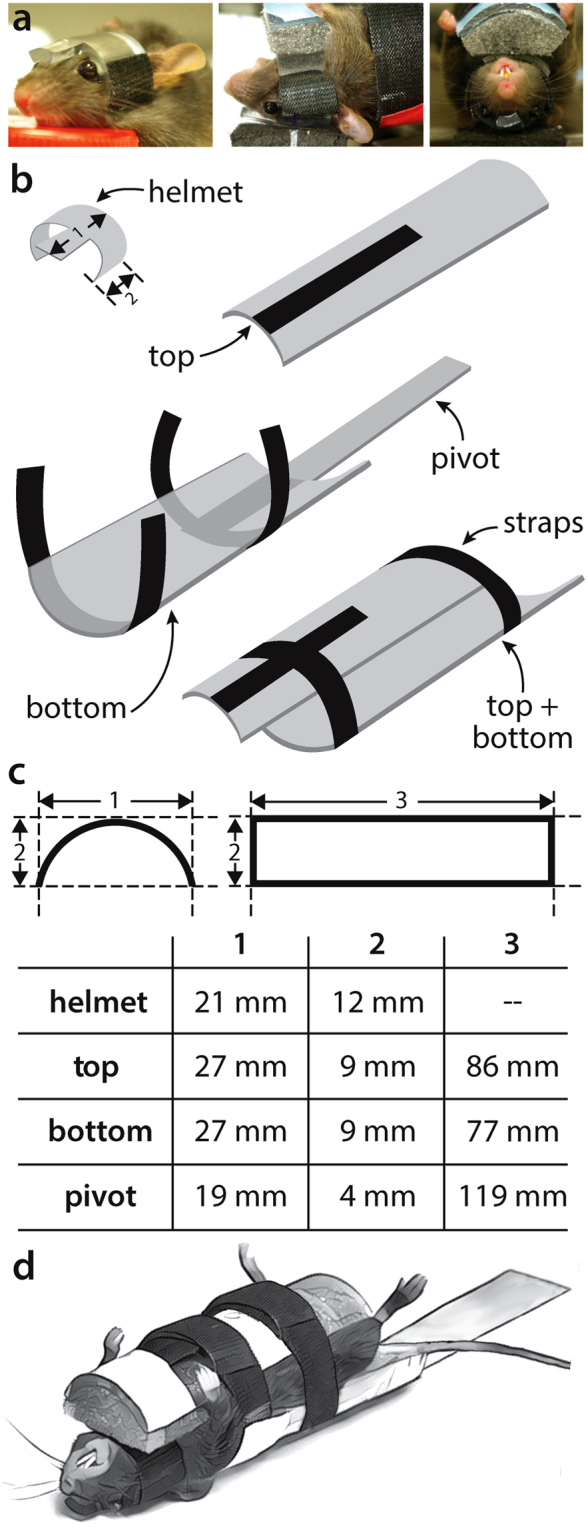
Helmet and cradle assembly. Representative images of mouse with semi-rigid helmet attached (left panel), and *in situ* views of positioning from lateral (middle panel) and front (right panel) (a). Schematic of helmet and cradle components with Velcro® strapping (**b**). Dimensions of helmet and cradle components (**c**). Illustration of mouse and helmet secured in cradle (**d**).

**Figure 2:**
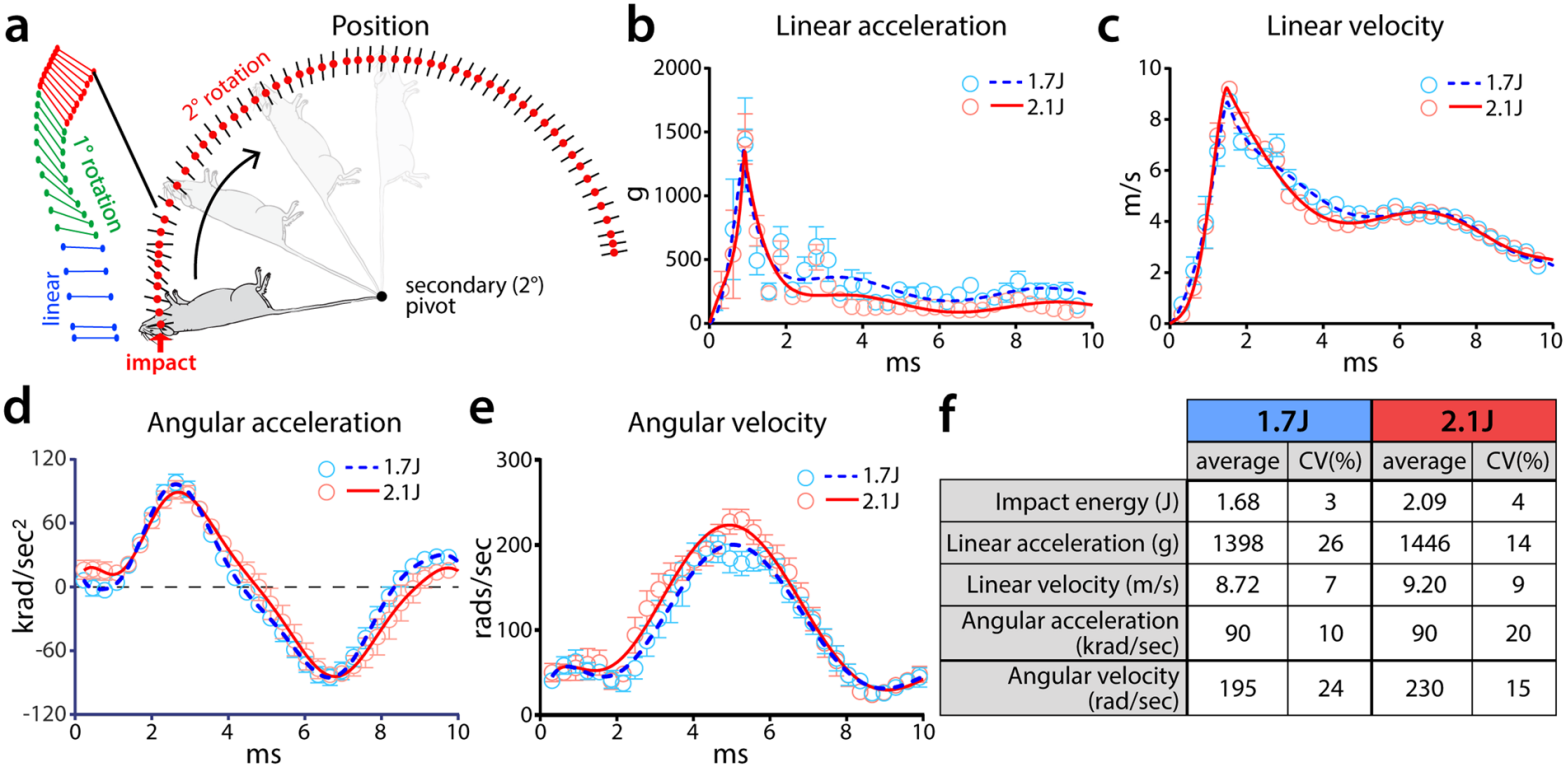
Kinematic analysis of modCHIMERA. Schematic representation of head and body movement following impact (**a**; helmet/cradle not shown for clarity). Colored lines connect tracking points illustrating the first 7.5 ms post-impact for a representative animal. Blue lines indicate initial phase of primarily linear head movement. Green phase includes the phase of rapid primary rotation of the head about the shoulders. Red lines represent the end of linear and primary rotation, and the continuation of secondary rotation about the pivot. Secondary rotation further tracked by red circles. Black lines represent angle of the head. Linear acceleration (**b**) and velocity (**c**) as function of time. Angular acceleration (**d**) and velocity (**e**) as a function of time. Kinematic outputs at 1.7 J and 2.1 J (**f**). Both impact intensities result in similar observed peak linear and angular motion of the head. Data represented as mean ± SEM. n = 9/group.

modCHIMERA is based on the Closed-Head Impact Model of Engineered Rotational Acceleration (CHIMERA) system, a pneumatic impact device that delivers a nonsurgical, tunable impact of monitored energy that is biomechanically consistent with mild TBI and permits unrestrained movement of the head^24^. Our goal in developing modCHIMERA was to adapt this platform to model more severe thresholds of injury, specifically complicated-mild and moderate TBI, while capitalizing on the system’s inherent strengths. Two significant modifications were required to achieve this goal: (1) protection of the skull from fractures through the use of a semi-rigid helmet and (2) protection of the spine and viscera from injury with a foam-lined rigid cradle that semi-restrains spine flexion (Fig. 1). A secondary pivot point was furthermore added, with the animal and cradle rotating about this point (Fig. 2a and Supplementary Movie 1).

We tested these modifications at two energy levels: 1.7 J and 2.1 J, impact energies that are 3.4-fold (1.7 J) and 4.2-fold (2.1 J) greater than initially reported for CHIMERA^24^. Without the above protective devices and modifications, mortality at these energies was close to 100%. Their use, however, resulted in a mortality rate of 8.8% and 23.4% at 1.7 J and 2.1 J, respectively (Supplementary Table 1). We further excluded animals with distracting injuries that were obvious on awakening from anesthesia (e.g., prominent hemiparesis, an almost invariable sign of skull fracture; see below), yielding overall inclusion rates of 84.1% (1.7 J) and 58.5% (2.1 J). Most animals (84% at 1.7 J and 94% at 2.1 J) experience some degree of ineffective respirations after injury, which was managed with lateral fingertip chest percussion until respiratory drive had recovered (38 ± 3.5 seconds), or for 1 minute if effective respiratory drive did not recover sooner.

**Figure 3:**
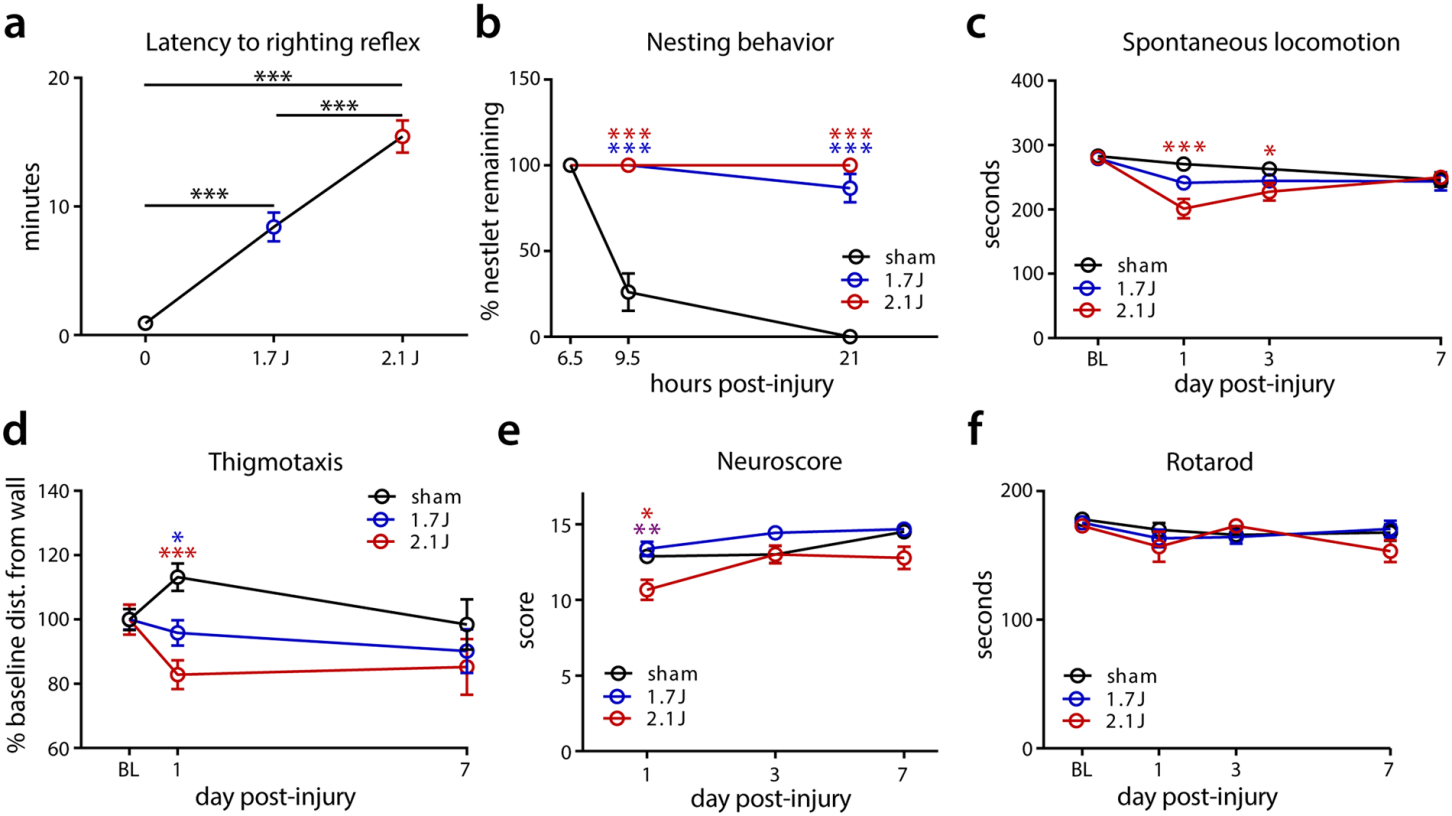
Acute functional deficits in modCHIMERA. Increased impact intensities result in longer latency to righting reflex (**a**; sham n = 14, 1.7 J n = 6, 2.1 J n = 14). Animals injured at 1.7 J and 2.1 J do not perform stereotypical nest building acutely post injury (**b**; sham n = 6, 1.7 J n = 10, 2.1 J n = 3). 2.1 J impacts result in significantly reduced acute spontaneous activity (**c**; sham n = 24, 1.7 J n = 12, 2.1 J n = 24). Thigmotaxic exploration is enhanced after modCHIMERA-induced TBI (**d**; sham n = 24, 1.7 J n = 12, 2.1 J n = 24). Neuroscore testing reveals impairment at 1 dpi in animals injured at 2.1 J (**e**; 1 dpi sham n = 8, 1.7 J n = 9, 2.1 J n = 11; 3 dpi sham n = 8, 1.7 J n = 9, 2.1 J n = 7; 7 dpi sham n = 8, 1.7 J n = 9, 2.1 J n = 3). Rotarod testing over first week post-injury reveals no effect at either 1.7 J or 2.1 J (**f**; 1 dpi sham n = 8, 1.7 J n = 8, 2.1 J n = 10; 3 dpi sham n = 8, 1.7 J n = 8, 2.1 J n = 7; 7 dpi sham n = 8, 1.7 J n = 8, 2.1 J n = 3). Data represented as mean ± SEM. Blue * highlights statistical significance of comparisons between control and 1.7 J modCHIMERA, red * between control and 2.1 J modCHIMERA, and purple * between 1.7 J and 2.1 J modCHIMERA. Rotarod, spontaneous locomotion, and neuroscore analyzed with the Kruskal-Wallis test followed by Dunn’s post-test. Latency to righting reflex and nesting behavior analyzed with two-way repeated measures ANOVA followed by Holm-Bonferroni post-hoc test.

With these adaptations, skull fractures are rare at 1.7 J, but are the most common exclusionary factor at 2.1 J, occurring in 18% of animals. Such fractures typically present as diastasis of the lambdoid suture causing subdural/subarachnoid hemorrhage near the cerebellum and unilateral paresis that is easily identified post-injury. Cortical lacerations generally underlie such fractures (Supplementary Table 1). Subdural hemorrhages were also occasionally noted, but were rarer and did not differ between energy levels significantly (Supplementary Table 1). There was no evidence of functionally-significant spinal cord injury, either on immediate post-injury observations of hind limb function or on functional assessments dependent on spinal pathways (see Fig. 3e,f). On careful inspection of a subset of animals, a minimal amount of blood was rarely observed in the spinal canal (4.4% and no more common at 2.1 vs 1.7 J).

### Kinematic analysis

To characterize inertial forces in modCHIMERA, high-speed videography (3200 fps) was used for kinematic analysis of head movement in the first 10 ms after impact. Three broad kinematic parameters were investigated at 1.7 J and 2.1 J (n = 9/energy level): linear motion of the head, primary rotation of the head about the shoulders, and secondary rotation about the secondary pivot (Fig. 2a and Supplementary Movie 1). Following impact, the head first moves vertically (Fig. 2a, blue lines) followed by a ~2 ms period of primary rotation about the shoulders in the sagittal plane (Fig. 2a, green lines), and finally a slower ~200 ms phase of rotation about a secondary pivot (Fig. 2a, red lines; also see Supplementary Movie 2), also in the sagittal plane.

Peak linear acceleration occurs at 1 ms, reaching 1398 g (1.7 J) and 1446 g (2.1 J) (Fig. 2b,f). Peak linear velocity occurs at 1.5 ms post-impact and reaches 8.72 m/s (1.7 J) and 9.20 m/s (2.1 J) (Fig. 2c,f). Angular acceleration peaks at 90 krad/sec^2^ (1.7 J and 2.1 J) and is reached at 2.5 ms post-impact (Fig. 2d,f). Peak angular velocity occurs at 5 ms, reaching 195 rad/sec (1.7 J) and 230 rad/sec (2.1 J) (Fig. 2e,f). Following a 175° rotation about the secondary pivot, the animal comes to rest on a cushioned platform. The coefficient of variation for all kinematic parameters is tightly constrained (7–26%), indicative of a consistent and reliable biomechanical profile (Fig. 2f). Interestingly, despite a 24.4% greater impact energy at 2.1 J vs. 1.7 J, kinematic parameters were only modestly increased at the higher energy level, implying a more substantial increase in impact as opposed to inertial loading (see below for discussion of likely contributions of these factors).

At 1.7 J modCHIMERA imparts an impact energy that is 3.4× that initially reported for the CHIMERA model^24^, increasing to 4.2× at 2.1 J. Resultantly, peak linear velocity of the head is 1.32 × (1.7 J) and 1.39 × (2.1 J) greater and linear acceleration is 3.63 × (1.7 J) and 3.75 × (2.1 J) greater. However, angular velocity and acceleration are both lower than previously reported^24^ (0.64× at 1.7 J and 0.75× at 2.1 J), resulting from constraints conferred by the cradle to avoid spinal and visceral injury. We employed the method of equal stress-equal velocity^24–26^ to scale injury biomechanics to human TBI^27,28^. Per this method a scaling factor of 13.8^24^ was applied to linear and angular velocity to account for size differences between the mouse and human brain, while rotational acceleration was scaled using the square of this factor. Linear velocity is not scaled^26^. Per this analysis, peak linear velocity and acceleration reach the range of average to severe human-equivalent sport concussions (Supplementary Table 2)^26,29,30^. Rotational velocity and acceleration, important determinants of pathology in humans and larger animal models, however, remain below this scaled threshold^26,29,30^.

### Short-term functional and behavioral deficits

Animals exhibited a number of short-term deficits following modCHIMERA-induced TBI (Fig. 3). The latency to righting reflex (LRR), a surrogate measurement for loss of consciousness after brain injury, was significantly prolonged immediately following injury in a severity-dependent manner (Fig. 3a). At the 1.7 J energy level, LRR was 8 ± 1.1 minutes vs. 0.9 ± 0.11 min for controls; this increased to 15 ± 1.3 minutes at 2.1 J with a range extending to 25 min. Although LRR has been used as a means to stratify injury severity into mild, moderate, and severe categories for the purpose of comparing to human TBI^21^, model-dependent effects preclude the application of strict cut-off values for each level of severity. Nonetheless, LRR times averaging 8 to 15 min generally align with injuries at the severe end of a mild to a moderate TBI^21^.

Signs of disrupted spontaneous behaviors were observed for three days after injury at both intensities. Injured animals exhibited drastically-reduced nesting behavior in the nestlet test at both time points investigated (Fig. 3b). Consistent with this loss of stereotypical behavior, a significant decrease in spontaneous exploration was noted at 1 and 3 dpi for animals injured at 2.1 J vs. controls (Fig. 3c). A similar trend was observed at 1.7 J that did not reach statistical significance (*p* = 0.09). Activity recovered to sham levels by 7 dpi. At 1 dpi, animals subjected to modCHIMERA at both energy levels remained closer to the walls of the testing chamber compared to control animals, a sign of increased anxiety (Fig. 3d). Reduced spontaneous activity at this time point at 2.1 J, however, potentially confounds conclusions about acute anxiety-related behaviors at this energy level.

A composite neuroscore specifically assessing strength, balance, and coordination revealed decreased performance 1 dpi at 2.1 J (Fig. 3e). These deficits subsequently improved, with injured animals performing similar to controls at 3 dpi. No statistically-significant differences in neuroscore were detected at 1.7 J vs. controls. Rotarod testing revealed no significant differences in motor coordination (Fig. 3f). Taken together, TBI induced by modCHIMERA resulted in substantial acute impairment in spontaneous function and behavior, but more modest deficits in stimulated tests (NS and RR).

### Cell death

**Figure 4:**
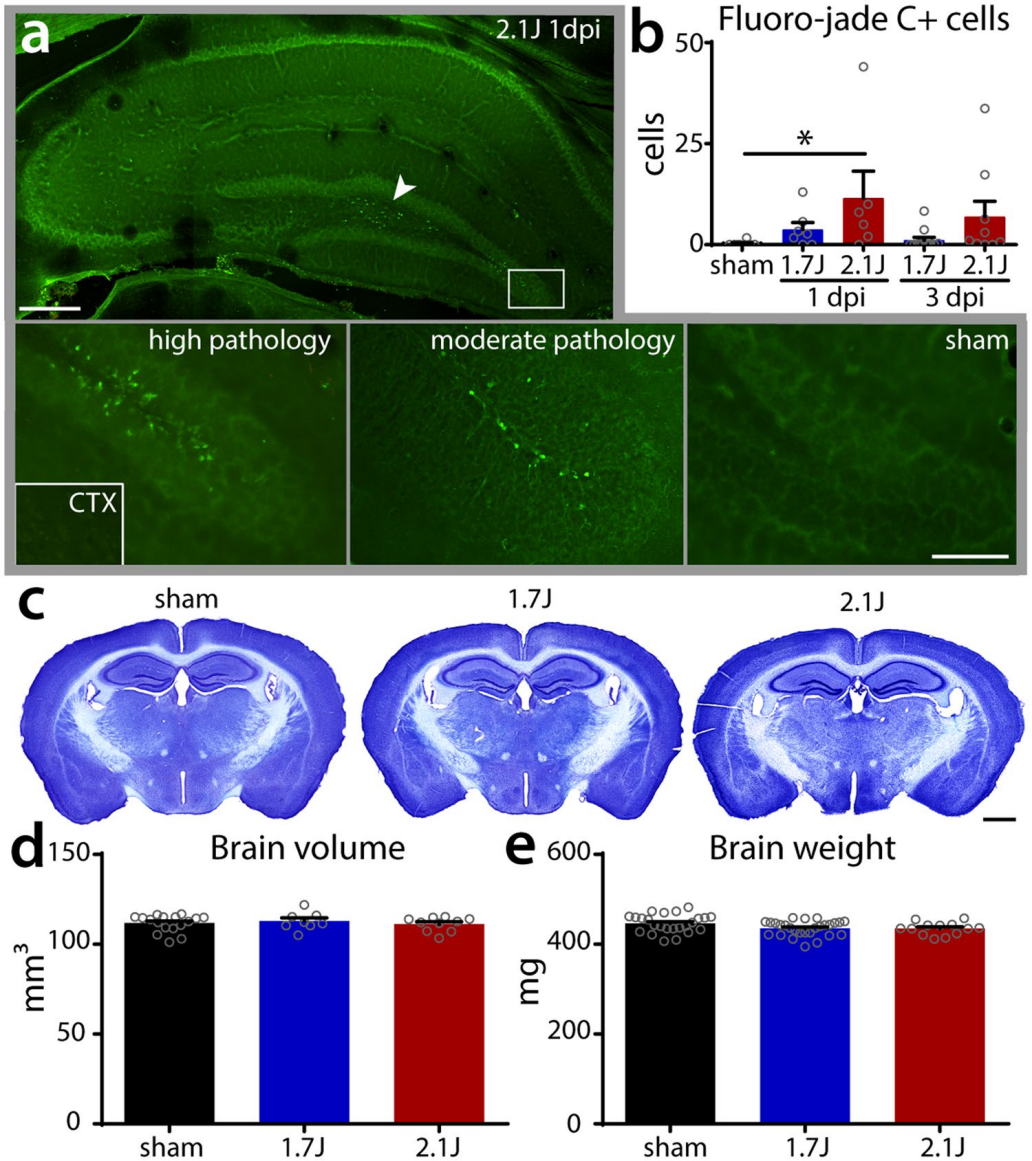
Cell death and lack of atrophy after modCHIMERA. Fluoro-jade C positive cells in the hippocampus after 2.1 J injury (**a**) are found in the dentate gyrus (panels below) and hilus (arrowhead), but not in cortex (CTX; from high-pathology animal; same magnification). Quantification of fluoro-jade C+ cells in hippocampus across injury groups at 1 and 3 dpi reveals significant cell death at 1 dpi in animals injured at 2.1 J (**b**; 1 dpi sham n = 8, 1.7 J n = 7, 2.1 J n = 6; 3 dpi 1.7 J n = 12, 2.1 J n = 9). The brain remains grossly intact at one-month post-injury at both energy levels (**c**; cresyl violet stain). Quantification of brain volume (**d**) and brain weight (**e**; sham n = 24, 1.7 J n = 27, 2.1 J n = 13) at one-month post-injury reveal no differences between injured animals and controls. Data represented as mean ± SEM. Fluoro-jade C counts, brain volume, and brain weight analyzed with Kruskal-Wallis test followed by Dunn’s post-test. Scale bar (**a**) upper panel 325 µm; lower panels 100 µm; (**c**) 1 mm.

Cell death is encountered to varying degrees in published TBI models, but is most prominent after contusional, impact-driven injuries such as those produced by lateral fluid percussion injury or controlled cortical impact^23^. To investigate cell death following modCHIMERA, we labeled brain sections at 1 and 3 dpi using fluoro-jade C, a histochemical stain that readily identifies degenerating neurons. Fluoro-jade C+ neurons were absent in control animals, but were identified in the hippocampal dentate gyrus and hilus bilaterally after modCHIMERA injury (Fig. 4a). Quantification of total hippocampal fluoro-jade C+ neurons revealed a statistically-significant increase neuronal death at 2.1 J, though a smaller degree of neuronal death was also observed at 1.7 J in the same locations (Fig. 4b, *p* = 0.0542). Neuronal death was less apparent 3 dpi, implying a monophasic process over this interval. Fluoro-jade C+ neurons were not detected in the cortex (Fig. 4a, CTX inset) or in any other brain structures in the absence of cortical damage in animals excluded due to skull fractures (see Supplementary Table 1).

To determine whether subacute neuronal death or significant loss of non-neuronal cells might occur after modCHIMERA, we measured cerebral volume at 1-month post-injury (Fig. 4c). Quantification of brain volume across 3 mm of tissue in the rostral-caudal axis centered around the impact site revealed no significant differences in brain volume across groups (Fig. 4d). Consistent with this, there were no differences in brain weight 1-month post-injury (Fig. 4e). Thus modCHIMERA induces modest levels of neuronal death in the hippocampus bilaterally but no clear diffuse atrophy.

### White matter injury

**Figure 5:**
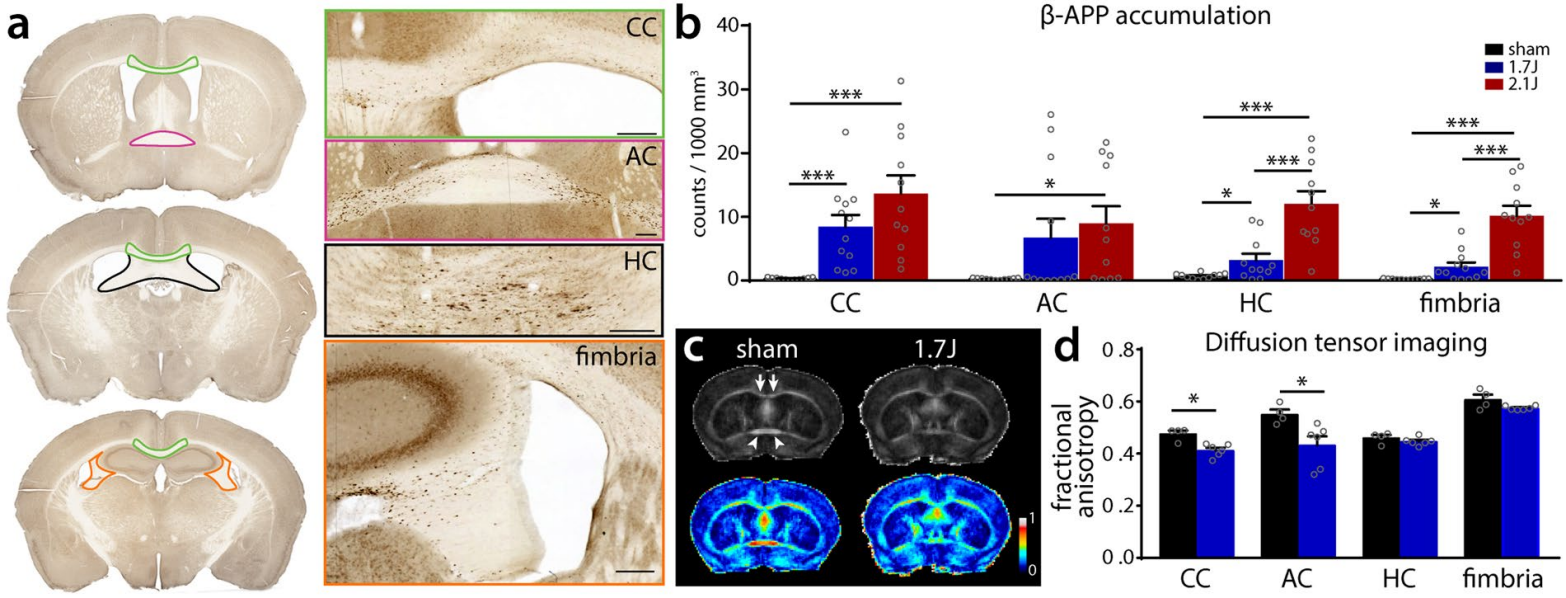
Histological and radiological white matter injury after modCHIMERA. β-APP staining and identification of ROIs for corpus callosum (CC), anterior commissure (AC), hippocampal commissure (HC), and fimbria following modCHIMERA (**a**). Note punctate accumulation of β-APP in all regions. Quantification of β-APP puncta at 1.7 J and 2.1 J compared to controls in these regions (b; sham n = 11, 1.7 J n = 12, 2.1 J n = 11). Animals injured at 2.1 J exhibit significant axon injury compared to controls in all four regions. Animals injured at 1.7 J exhibit significant axon injury in the CC, HC, and fimbria compared to controls. Fractional anisotropy maps (color LUT applied in lower panels) following 1.7 J modCHIMERA (c; arrowheads identify anterior commissure; arrows identify corpus callosum). Note reduced anisotropy in these structures following modCHIMERA. Quantification of fractional anisotropy across the same regions analyzed with β-APP staining reveals significant axon injury in the CC and AC (d; sham n = 4, 1.7 J n = 6). Data represented as mean ± SEM. β-APP counts in anterior commissure analyzed with Kruskal-Wallis test followed by Dunn’s post-test. β-APP counts in corpus callosum/fimbria/hippocampal commissure tested by one-way ANOVA followed by Holm-Bonferroni post-hoc test. Regional fractional anisotropy quantification analyzed by Mann-Whitney U test with correction for multiple comparisons. Scale bar (**a**) 200 µm. n = for β-APP analysis; 4–6 for MRI analysis.

White matter damage is a strong predictor of neurological deficits after TBI in humans^31,32^. We employed 2 histological assays and diffusion tensor imaging (DTI) to assess axonal injury following modCHIMERA. Staining against β-APP revealed evidence of axonal injury in multiple white matter tracts at 1 dpi including the corpus callosum, anterior commissure, hippocampal commissure, and fimbria bilaterally (Fig. 5a). Quantitative analysis of β-APP+ puncta in these regions at 1.7 J and 2.1 J demonstrated an injury intensity-dependent amount of axonal injury (Fig. 5b). To corroborate these findings, we assayed for cytoskeletal injury using SMI-31, an antibody targeted to phosphorylated neurofilament-H. SMI-31+ puncta were observed in the same regions as β-APP in injured animals (Supplementary Fig. 3).

Evaluating radiological biomarkers of pathological changes is a critical translational goal in brain injury research. DTI is a well-validated MRI technique that can reveal axonal injury in white matter structures^33,34^. In agreement with β-APP quantification, DTI of *ex vivo* brains 2 days after 1.7 J modCHIMERA injury revealed multiregional decrements in fractional anisotropy (FA) consistent with axonal injury (Fig. 5c). Reduced FA reached statistical significance in the corpus callosum and anterior commissure (Fig. 5d). These results demonstrate that multifocal histological and radiological axonal injury is a feature of modCHIMERA.

### Microgliosis

**Figure 6:**
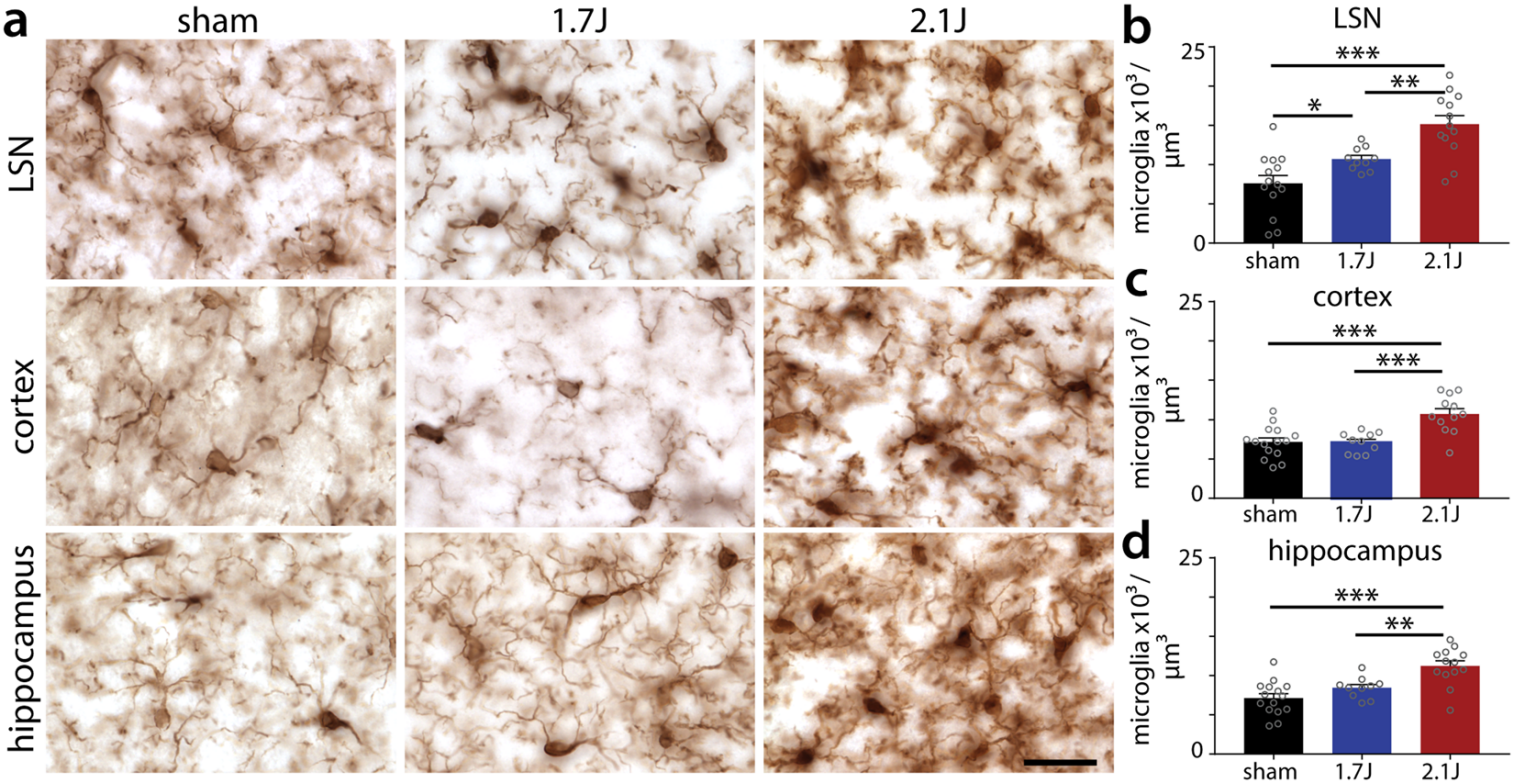
Multifocal microgliosis following modCHIMERA. Microglia in the lateral septal nucleus (LSN), cortex, and hippocampus as labeled with Iba1 staining at 30 dpi in controls and after 1.7 J or 2.1 J injuries (**a**). Quantification of microglial counts in these regions across groups reveals significant increases in microglial density in all regions following 2.1 J injuries (**b**–**d**). Animals injured at 1.7 J exhibit significant microglial accumulation in the LSN 30 dpi (**b**). Data represented as mean ± SEM. Microglial density analyzed by one-way ANOVA followed by Holm-Bonferroni post-hoc test. Scale bar 25 µm. Sham n = 15, 1.7 J n = 10, 2.1 J n = 13.

Neuroinflammation is linked with lasting neurologic impairment after TBI, even in the absence of focal tissue loss^35^. Microglia, the resident immune cells of the brain, are major contributors to this response^36^ and increase in number following brain injury. Immunostaining against Iba-1, a pan-microglial marker, at 1-month post-injury revealed a prominent increase in microglial density in several brain regions after injury at 2.1 J (Fig. 6a). Quantitative analysis of microglial density confirmed this increase in the lateral septal nucleus, isocortex, and hippocampus (Fig. 6b–d). At 1.7 J the effect was less pronounced, though significantly more microglia were found in the lateral septal nucleus vs. controls (Fig. 6a,b). Thus modCHIMERA induces microglial neuroinflammation at both energy levels, however this response is more widespread at 2.1 J.

### Distribution of pathology

Careful inspection of sections immunolabeled against Iba-1 and β-APP revealed subtle patterning of injury across the brain. Evidence of a locally-enhanced microglial response and neuritic injury was observed in superficial layers of cortex in stereotyped locations (Supplementary Fig. 4a,b): Subtle evidence of microscopic injury was identified in the bilateral entorhinal area/lateral isocortex in all injured animals at both impact energies. A similar neuroinflammatory and neuritic response was noted in the olfactory tubercle/piriform cortex in 62.5% of animals at 1.7 J, and 71.4% at 2.1 J. Pathology in these regions was characterized by an increased prominence of microglia with altered branching patterns and focal accumulation of β-APP. In 50% of animals at both energy levels, a small, superficial cortical defect was observed in cresyl violet stained sections in one of these stereotyped locations, accompanied by localized disruption of the BBB (Supplementary Fig. 4c). The observed pattern of injury, limited to the superficial cortex, implies that a brain-skull or brain-meninges interaction underlies this patterning. Interestingly, outside of these small cortical injuries, no group-wise differences in vascular integrity were detected at either energy level (Supplementary Fig. 5). A rim of meningeal iron accumulation, however, was observed in a subset of injured animals (35% at 1.7 J, 53% at 2.1 J) that was not seen in controls (Supplementary Fig. 5d), suggestive of modest and transient subarachnoid bleeding.

### Neurobehavioral deficits

Neurocognitive impairment after TBI is often disabling and persistent, and affects several neuropsychiatric domains^37–39^. Therapeutic preclinical testing with the goal of improving long-term outcomes after TBI ideally includes such end points. We therefore investigated a battery of neurobehavioral outcomes focused on learning, memory, anxiety- and depressive-like behaviors, and socialization/social memory between 2 and 4 weeks after modCHIMERA at 1.7 J and 2.1 J (see Supplementary Fig. 1 for time line of behavioral testing).

**Figure 7:**
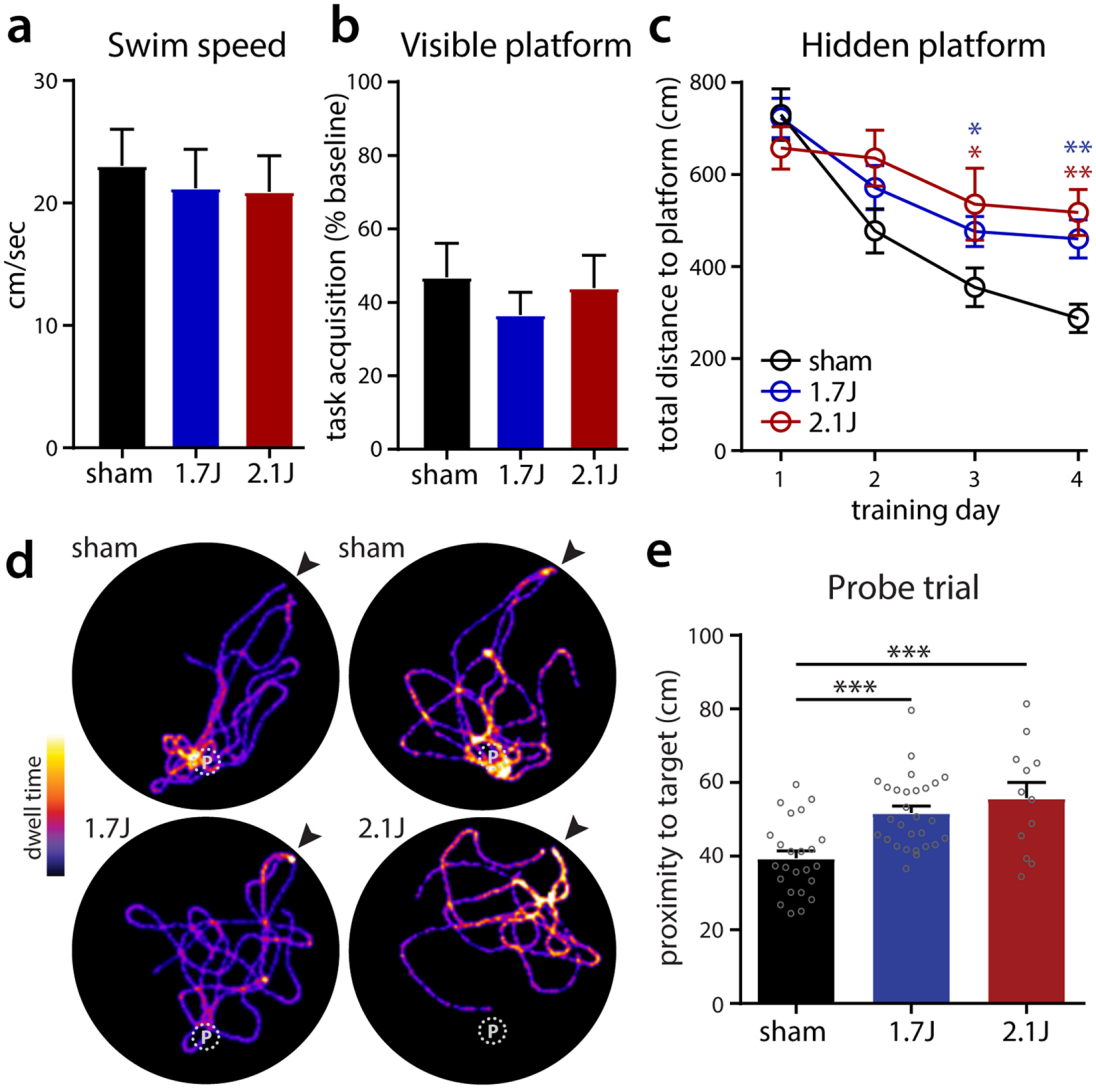
Spatial learning and memory are impaired after modCHIMERA. Swim speed comparison during visible platform testing in shams and injured animals (**a**) demonstrates similar performance across groups. Improvement in total distance to target over 3 days of visible platform testing (**b**) demonstrates similar ability to acquire task in all groups. Total distance to platform during four days of hidden platform testing reveals injured animals travel further before reaching the platform (**c**). Heat maps depicting dwell time over 15 seconds of probe testing depict reduced performance of injured animals to navigate directly to the previous location of the hidden platform (**d**). Arrowhead indicates insertion point; location of platform indicated by P. Performance of injured animals is depicted below their cohort-matched controls. Quantification of average proximity to target during probe testing (**e**) reveals reduced performance after injury following either 1.7 J or 2.1 J injuries. Data represented as mean ± SEM. Swim speed and probe trial results analyzed by one-way ANOVA followed by Holm-Bonferroni post-hoc test. Visible platform testing analyzed with the Kruskal-Wallis test followed by Dunn’s post-test. Hidden platform testing analyzed by two-way repeated measures ANOVA followed by Holm-Bonferroni post-hoc test. Sham n = 24, 1.7 J n = 28, 2.1 J n = 12.

Animals were assessed for deficits in learning and memory using the Morris water maze (MWM)^40^ test starting at 2 weeks post-injury. Animals were first assessed with 3 days of visible platform testing on 14 to 16 dpi to evaluate baseline swim speeds and ability/motivation to acquire the task. There were no group-wise differences in these endpoints (Fig. 7a,b). Starting 19 dpi animals underwent 4 consecutive days of hidden platform training to evaluate spatial learning. Injured animals at both energy levels performed significantly worse in this task compared to controls (Fig. 7c), indicating a long-lasting deficit in spatial learning following modCHIMERA at both energy levels. The day following completion of hidden platform training (23 dpi) animals underwent a single probe trial in which the platform was removed and proximity to the platform’s prior location was monitored. Heat maps of location in the pool demonstrated clear differences in the ability to quickly navigate to the platform location, with sham-injured animals performing consistently better than animals injured at 1.7 J or 2.1 J (Fig. 7d). Quantification of target proximity confirmed these observations (Fig. 7e). Interestingly, there were only modest differences between 1.7 J and 2.1 J in both hidden platform and probe trial performance, despite substantially greater acute functional impairment and neuropathology at 2.1 J (see Figs 3–5) including hippocampal neuronal death (though kinematic profiles were notably similar; see Fig. 2). This suggests that acute functional assays are poor surrogates for longer-term learning and memory deficits, and that unmeasured pathological pathways are likely important in the generation of these deficits.

We further assessed chronic anxiety-related behaviors using the elevated plus maze and thigmotaxis in the open field test at 26 dpi, and depressive-like behaviors with the tail suspension test at 30 dpi. No group-wise differences were noted between controls and injured animals in any endpoints in these tests (Supplementary Fig. 6), indicating that modCHIMERA results in minimal lasting emotional impairment.

**Figure 8:**
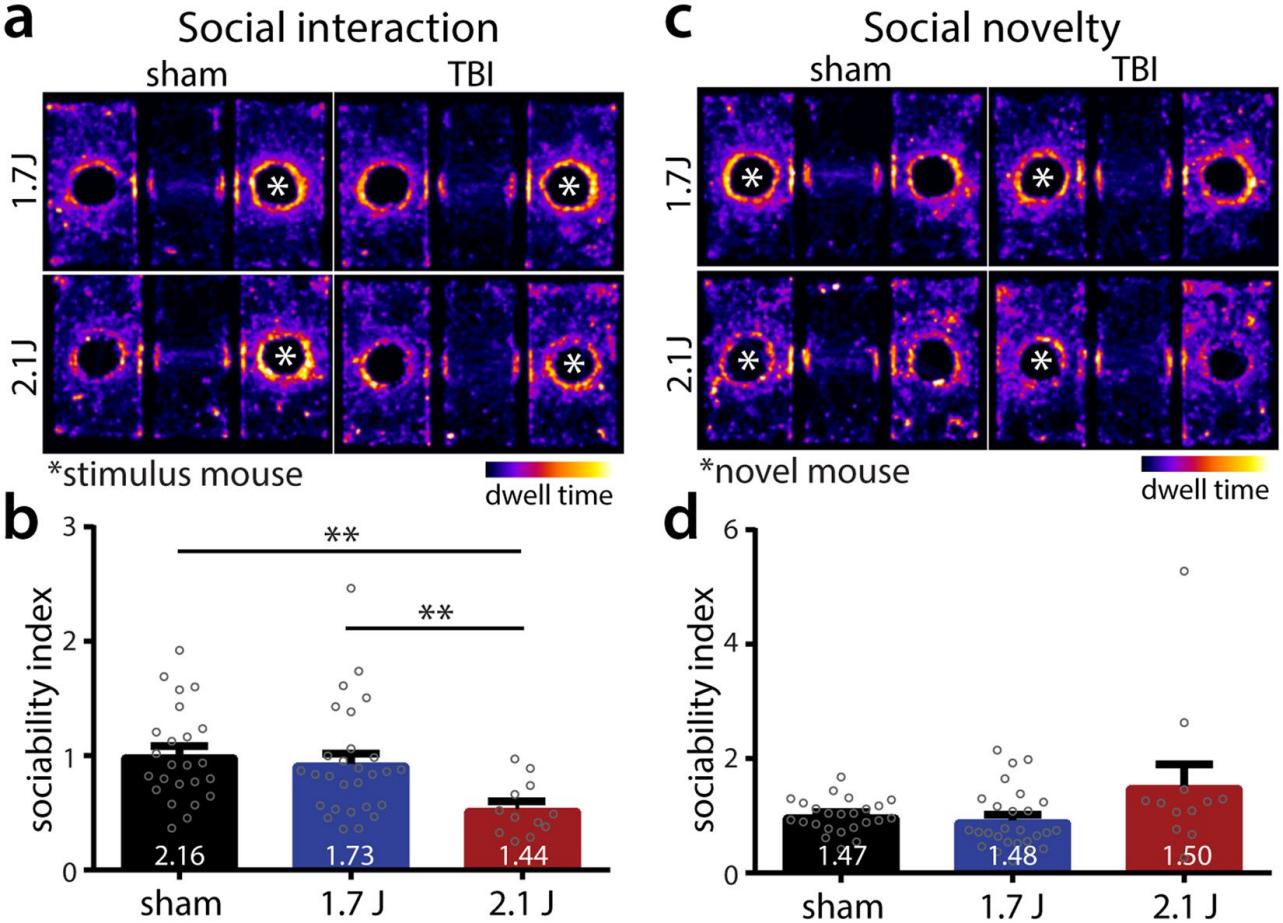
Socialization deficits after modCHIMERA. Heat maps depicting dwell time in the three-chamber social interaction apparatus during social interaction testing (**a**; * indicates location of the stimulus mouse). Cohort-matched controls are depicted to the left of injury groups. Note controls show greater preference for stimulus mice compared to injured animals. Quantification of social interaction reveals animals injured at 2.1 J are less sociable than controls or animals injured at 1.7 J (**b**). Ratio of time interacting with stimulus mouse to time interacting with dummy mouse listed at bottom of bar for each group. Heat maps depicting dwell time for social novelty testing (**c**; * indicates location of the novel mouse). Quantification of social novelty seeking reveals no difference between any of the groups (**d**). Ratio of time with novel mouse to time with original stimulus mouse listed at bottom of bar for each group. Sociability index is ratio of time with stimulus to dummy mouse normalized to within-cohort controls (see Methods). Data represented as mean ± SEM. Social interaction and social novelty analyzed by one-way ANOVA followed by Holm-Bonferroni post-hoc test. Sham n = 24, 1.7 J n = 28, 2.1 J n = 12.

Relationship dysfunction can be prominent and disabling after TBI^41^. To evaluate social behavior, we subjected animals to a 3-chamber social interaction and social novelty test at 27 dpi. Animals injured at 2.1 J exhibited a significant reduction in social interaction compared to sham-injured animals or animals injured at 1.7 J (Fig. 8a,b). While sham animals spent over twice as much time with the stimulus mouse compared to a dummy mouse, animals injured at 2.1 J spent only 44% more time with the stimulus mouse (Fig. 8b). Animals injured at 1.7 J demonstrated an intermediate preference, spending only 73% more time with the stimulus mouse, but this did not reach statistical significance. No statistically-significant differences were found in social interaction between sham animals and those injured at 1.7 J. There were no statistically-significant group-wise differences in social novelty behavior, with all groups exhibiting a moderate preference for the novel compared to the initial mouse (Fig. 8c,d). There were no differences in performance in an olfactory stimulus test between groups (olfaction required for normal social behavior; data not shown). Considered together these data demonstrate multidomain neurobehavioral deficits following modCHIMERA persistent to 1-month post-injury. To preliminarily assess sex-related differences in modCHIMERA, we also performed a battery of neurobehavioral and histological assessments in female animals (Supplementary Figs 7 and 8).

To assess the consequences of the small cortical lesions observed with cresyl violet staining in a subset of injured animals, we compared functional and behavioral outcomes in injured animals with and without such defects across the tests reported above. Interestingly, we found no significant differences between these groups in acute neuroscore, spontaneous activity, thigmotaxic exploration (Supplementary Fig. 9a–c), performance in the MWM (Supplementary Fig. 9d; 23 dpi), or performance in social interaction tests (Supplementary Fig. 9e; 27 dpi). Thus the effect of modCHIMERA-induced TBI on acute and long-term neurobehavioral outcomes is likely driven instead by diffuse/multifocal processes.

## Discussion

We report an adaptation of the CHIMERA model of mild TBI that permitted scaling to more severe thresholds while preserving the reliability, tuning, and monitoring of input forces that are inherent strengths of this platform. We extensively characterized modCHIMERA to evaluate its suitability for examining various pathological and neurobehavioral endpoints. modCHIMERA exhibits multifocal histological and radiological axonal injury. Microgliosis is multifocal, though less prominent at 1.7 J compared to 2.1 J, and cell death is limited to the hippocampus. Injured animals demonstrate deficits in learning and memory at least to 3 weeks post-injury. Social behaviors are furthermore impaired after 2.1 J injuries at 1-month, while emotional dysregulation (anxiety- and depressive-like behavior) was not detected with the assays employed. Animals exhibited subtle signs of cortical injury at stereotyped locations suggestive of anatomically-constrained interactions between the brain and skull or meninges during injury.

Adaptations to limit extracerebral injuries yielded low mortality and exclusion rates (11.4% and 20.5% at 1.7 J) at 3.4-fold greater energy than initially reported for this system. At yet higher energy levels (2.1 J, 4.2-fold greater energy) mortality remained in line with that reported in several injury models (23.4%)^42,43^, but sub-lethal skull fracture became more common (18%).

TBI has been called perhaps the most heterogeneous of neurological disorders^44^. No one model can capture every aspect of the human condition. Existing models have been divided along several axes including severity of injury, surgical vs. non-surgical, closed head vs. open, diffuse vs. focal, and impact vs. inertial loading^21,23,45,46^. modCHIMERA is not the first model to achieve any of these ends, rather it straddles several boundaries thereby occupying a somewhat rare position in this parameter space, as well as offering a number of practical strengths. Specifically, modCHIMERA straddles the complicated-mild/moderate TBI boundary (but see below), and entails impact followed by semi-retrained linear and rotational acceleration. Interestingly, it also appears to induce both diffuse and multifocal injury, a common aspect of complicated-mild to severe human TBI. Practical advantages of the model include complete lack of surgical procedures, a rapid workflow (5–7 min/animal), and the ability to precisely tune and monitor impact energy.

The targeting of specific human TBI severity levels with rodent models is fraught with important caveats. First, human TBI severity determinations are not fully standardized (for example see^6–10^) and are based on factors that translate poorly to rodent models, for example the Glasgow coma scale and CT scan abnormalities. Clinical TBI classifications (mild, moderate, and severe) may furthermore not be ideal targets in terms of stratifying patients by outcome or pathophysiology, as studies of complicated-mild TBI suggest that outcomes from such injures are more similar to those after moderate TBI^12^, and intracranial lesions are much more common in complicated-mild TBI and moderate TBI vs. mild TBI^47^. In the absence of directly comparable acute classifiers in rodent TBI, lateral righting reflex (LRR) has frequently been used as a severity discriminator (for review see^21^). There are several challenges with using LRR to compare between models, let alone with humans, but a range of <15 minutes has been invoked for mild injuries, 15–20 minutes for moderate, and >20 minutes for severe^21^. By these criteria modCHIMERA falls on the severe end of a mild TBI to a moderate TBI. The finding of focal neuropathology in the entorhinal area/lateral isocortex is a further connection with complicated-mild and moderate TBI, the former of which is often defined by the presence of such radiographic injury^12–14,17^. Such lesions are less prevalent in mild TBI, occurring in only 20% of patients with GCS of 14 and dropping to only 5% of patients with GCS of 15^47^.

Despite these parallels, however, other models of moderate-severe injury are potentially more appropriate for specific scenarios or questions. For the analysis of focal injury, lateral fluid percussion TBI and controlled cortical impact models both induce tunable, focal gray and white matter injury, and are reliable and reproducible along several pathological and neurobehavioral outcomes. Elements of diffuse injury have also been reported with these models, though this appears to be less extensive than that observed with modCHIMERA^48,49^. Fluid percussion and controlled cortical impact models do not include inertial elements, and they are much lower throughput as they generally require substantial surgical preparations.

Weight drop models can induce diffuse injury at a severity level that appears similar to modCHIMERA. However, weight drop models exceeding mild TBI thresholds generally involve surgery^21,23^. They have furthermore been difficult to tightly constrain, partly as a result of frictional forces and a poorly-controlled second impact, though some variants eliminate this second hit^50^. Motion after impact is also traditionally very limited in weight-drop models, as the animal rests on a semi-rigid material. Several more recent variants now permit less-restrained motion through the use of low tensile platforms through which the animal falls after impact^23,50,51^. Detailed kinematic analysis of such post-impact motion is, to our knowledge, currently lacking, as are neurobehavioral characterizations beyond the acute period.

While modCHIMERA induces impact as well as linear and rotational inertial loading, it is unlikely all three factors contribute equally to outcomes. In particular, the small size of the mouse brain makes it difficult to impossible to model a biomechanically-meaningful, purely rotational injury on the scale of moderate human TBI. Adaptations necessary to protect the spine from injury in this model, moreover, resulted in a reduction in rotational forces compared with published CHIMERA kinematic parameters^24,25^.

Linear inertial loading reached scaled human injury thresholds. Humans and large animal models are, however, more susceptible to rotational acceleration^52^, which did not reach scaled thresholds. The lissencephalic rodent brain presents a further impediment to biomechanical modeling of human brain injury. Larger animal models (pigs, dogs, non-human primates) overcome this problem while also permitting rotational kinematics in the range of human injury.

The observation of significantly greater pathology after modCHIMERA at 2.1 J vs. 1.7 J despite minimal differences in kinematic parameters suggests that impact may be the biggest driver of injury in this model. The use of finite element modeling in combination with knowledge of the location of cortical injuries and the pattern of diffuse pathology in modCHIMERA may shed further light on the interplay between injury factors.

Several important limitations must be acknowledged regarding modCHIMERA. Behavioral outcomes were not evaluated at truly chronic (e.g., 6–12 months) time points. Therefore, it remains possible that modCHIMERA substantially delays recovery of learning, memory, and social behavior, but that injured animals ultimately recover to baseline. CHIMERA and the modifications needed to scale to modCHIMERA were designed to limit variability and improve reproducibility. The coefficient of variability in impact and kinematic parameters (Fig. 2), and scatter plots of histological and neurobehavioral endpoints suggest that to an extent this aim has been achieved. Whether reliability is maintained across operators, however, will require testing of modCHIMERA in other research groups. We did not fully assess cardiopulmonary physiology following injury with this model, thus we cannot rule out an interaction between primary neurological injury and secondary insults. Efforts are currently underway to further characterize gender-related factors in modCHIMERA. Limited testing of modCHIMERA with several transgenic lines does not reveal any systematic issues with genetically-altered mice, but this has not been formally evaluated.

Several additional future directions are worth considering, including the effect of other major sources of disability after complicated-mild TBI such as acute stress, migraine modeling with spreading depolarization and trigeminal sensitization^53,54^, sleep-wake cycle alterations^54^, and the effect of repeat injuries or physiological alterations (hypoxia^55^, hypotension, etc.). Future exploration of primary pathophysiological avenues should include a deeper study of the regional, temporal, and functional roles of microglia and microglial activation in complicated-mild TBI/moderate TBI, and of the structural changes that underlie circuit disruption and functional network alterations after trauma.

In conclusion, modCHIMERA is a rapid, non-surgical model of murine closed-head injury characterized by axonal injury, neuroinflammation, hippocampal cell death, and multidomain neurobehavioral deficits. modCHIMERA exhibits acute severity metrics and injury patterns most consistent with a complicated-mild or moderate TBI, an understudied portion of the TBI severity spectrum that results in substantial morbidity and mortality, yet presents therapeutic targets that are potentially more susceptible to intervention than for severe TBI. This model may therefore be useful for the dissection of mechanistic pathways active after TBI, and in the testing of therapeutics against such injurious processes.

## Methods

### Animals

All animal experiments were approved by the Washington University Institutional Animal Care and Use Committee and performed in accordance with relevant guidelines and regulations. 14–15 week old male C57/BL/6 J mice (Cat# 000664 Jackson Laboratory, Bar Harbor, ME, USA) were allowed to acclimate for 1–2 weeks prior to all studies with 12-hour light-dark cycles. Food and water was provided *ad libitum*. At 16 weeks of age mice were randomly assigned to either injury or sham groups.

### CHIMERA modifications and injury set-up

Brain injuries were performed using the CHIMERA platform^24^ with the following modifications (step-by-step protocol available upon request): The experimental animal was anesthetized with 5% isoflurane for 2.5 minutes followed by 2.5% isoflurane maintenance during positioning via nose cone. While anesthetized, a helmet made of Tygon S3 B-44-4 × tubing (5/16-inch inner diameter/½-inch outer diameter) was placed directly posterior to the eyes (Fig. 1). Two Velcro® patches (6 × 12 mm) were attached to the sides of the helmet with cyanoacrylate adhesive (Loctite, Rocky Hill, CT., USA, cat. #234790). The mouse was then placed in a supine position in a foam/plastic cradle (19 g fully assembled) attached to the CHIMERA device with the stage at a 10° angle.

The cradle was made of polycarbonate tubing (1-1/8-inch inner diameter, 1-1/4-inch outer diameter, 1/16-inch wall thickness, Rockwell hardness R73–78, Amazon, Seattle, WA, USA, cat. #TPC-125/24) and closed-cell polyethelene foam tubing (Everbilt, cat. # ORP11812). The top, bottom, and pivot polycarbonate pieces were machined to the dimensions in Fig. 1. The foam and polycarbonate pieces were connected with adhesive tape (3 M VHB, St. Paul, MN, USA). The foam for the cradle bottom was cut to 6.5 × 2.5 cm with the long axis along the main axis of the foam tubing. Its thickness was reduced to 0.5 cm by trimming away from the outer circumference with a razor blade. The foam was then ‘notched’ at one end with a razor blade so that its thickness was gradually reduced over 1 cm from 5 mm to 3 mm thick at the end, allowing the animal’s neck to rest in this notch. It was then attached to the inner circumference of the cradle bottom, lining its entire length.

An untrimmed piece of foam was attached to the inner surface of the top polycarbonate cradle component so as to line its entire length. The foam was then trimmed so that the most anterior 12 mm formed a level ‘step-off’ that was 1 cm in thickness at its thickest point. The remaining 74 mm of foam was trimmed flush with the bottom edge of the polycarbonate cradle top so as to fill its inner circumference entirely.

Four Velcro® straps were attached to the cradle to secure the animal during positioning and impact (Amazon, Seattle, WA, USA, cat. #91140). The first segment (3.5 cm) was attached at its midpoint to the foam step-off on the cradle top with double adhesive tape (3 M VHB, St. Paul, MN, USA) to engage the helmet. The remaining segments were attached with laboratory tape (VWR, Randor, PA, USA, Cat. # 89097). The second segment (15 cm) formed the anterior strap (see Fig. 1d), and was attached beneath the cradle bottom at the Velcro® segment’s midpoint, flush with the cradle edge nearest the impact site. The third segment (15 cm) formed the posterior strap, and was attached at its midpoint to the bottom of the pivot flush with its proximal edge (Fig. 1b). The fourth segment (3.5 cm) was attached to the cradle top lengthwise along its outer surface (Fig. 1b) to function as a connection point with the anterior strap once closed, preventing movement of the cradle top relative to the bottom.

To prepare the cradle, the pivot was first oriented so that it was in the animal’s sagittal plane on the stage of the CHIMERA device, flush with the distal stage edge (away from the impact site). The pivot was then secured to the stage with laboratory tape (VWR, cat. #89097, Randor, PA, USA; ~6 cm of tape extending onto the pivot and ~6 cm wrapping onto the bottom of the stage), allowing the pivot to freely rotate about the distal stage edge in the sagittal plane. The cradle bottom was then positioned proximally, overlapping the pivot and along the same axis, so that the notched foam end was flush with the nearest edge of the pad surrounding the impact site of the CHIMERA device (13 mm from the epicenter of impact). The cradle bottom was secured to the pivot with ~4 cm of laboratory tape (VWR, Randor, PA, USA, cat. #89097).

The anesthetized, helmeted animal was placed supine on the cradle bottom and the head positioned as close as possible to its final location over the impact site: 4 mm posterior to the lateral canthus of the eye at midline. The top of the cradle was then positioned over the mouse so that the ‘step-off’ was directly in line with the helmet (see Fig. 1d). The Velcro® straps on the step-off were attached to the sides of the helmet, followed by closure of the anterior and posterior straps. The arms of the animal were repositioned to extend outside of the cradle.

Once the animal was secured, the tape connecting the cradle bottom and pivot was released from the pivot so that the cradle could rotate and transit along its long axis. Precise adjustments were made to rostrocaudal, lateral, and rotational positioning to center the head over the impact site. The cradle bottom and pivot were then re-secured (tape reattached) and isoflurane was maintained at 2.5% for an additional 1 minute to ensure an equal plane of anesthesia for all animals. Isoflurane was removed for 30 seconds immediately prior to impact to minimize its contribution to post-injury unconsciousness. Control experiments demonstrated no response to toe pinch after 30 seconds off isoflurane.

### Injuries

Two impact intensities were tested with modCHIMERA: 1.7 J and 2.1 J. Air pressure was set to achieve these energies (8 psi for 1.7 J and 9.8–10 psi for 2.1 J). Prior to each injury set, at least three test impacts were performed with a rubber dummy to fine tune to the desired impact energy (J = ½ mass piston (kg) × velocity^2^ (m/s) where piston mass is 0.05 kg and velocity is monitored for each impact). Measured velocities were 8.13 m/s (coefficient of variation 2.18%) for 1.7 J and 9.10 m/s (coefficient of variation 2.09%) for 2.1 J. Following impact the mouse and cradle freely rotate ~180° coming to rest on a soft rubber-foam catch (Everbilt cat. #PI16RSS) attached to the CHIMERA device (Supplementary Movie 1). The mouse was then quickly removed from the cradle and assessed for any signs of ineffective respirations or apnea. When this was observed, lateral fingertip chest percussion was performed within ~10 seconds from impact at a rate of ~350 percussions/minute until effective breathing returned (38 ± 3.5 seconds). Eye lubricant was applied and the mouse was placed in a warm recovery chamber until awake.

### Kinematics

Video acquisition for kinematic analysis was performed with a FPS1000 digital camera (The Slow Motion Camera Company; St. Albans, Hertfordshire, UK) at a frame rate of 3230 fps. Two tracking points were added to the left side of the helmet. These points were manually isolated for each frame in Adobe Photoshop (Adobe, San Jose, CA, USA). To limit error, the isolation process was repeated three times independently for each video, with the average used for final measurements. SMART automated tracking software (v3.0 Panlab/Harvard Apparatus, Barcelona, Spain) was used to find the center of each tracking point in X/Y space.

Linear kinematic parameters (linear velocity and acceleration) for the center of the head were calculated from the average linear velocity and acceleration of each tracking point. For analysis of rotation, the X/Y coordinates obtained from SMART for each point were processed using MATLAB (MathWorks, Natick, MA, USA). First, linear kinematic parameters were calculated using the MATLAB Gradient (central difference) function, yielding the velocity and acceleration for each point. Exploiting the fact that the tracking markers represent two points on a nearly rigid body in 2D motion, the linear velocity and acceleration of both markers were then used to calculate the angular velocity of the head.

The equations describing the velocities of two points on a rigid body are:

1a $${{\rm{V}}}_{1{\rm{x}}}={{\rm{V}}}_{2{\rm{x}}}-{{\rm{\omega }}{\rm{\Delta }}}_{{\rm{Y}}},$$

1b $${{\rm{V}}}_{1{\rm{y}}}={{\rm{V}}}_{2{\rm{y}}}+{{\rm{\omega }}{\rm{\Delta }}}_{{\rm{X}}},$$

where [V_1x_,V_1y_] = velocity vector of point 1, [V_2x_,V_2y_] = velocity vector of point 2, [Δ_X_, Δ_Y_] = relative position vector of point 1 with respect to point 2, and ω = angular velocity. The value of ω that minimized the squared error in these equations was found, using the previously-estimated vectors of linear velocity and relative position. Calculations were performed in MATLAB using data from three successive frames to reduce the effects of noise. For the calculation of angular acceleration, the angular velocity data was first processed through a 10 Hz Butterworth filter^56^, and the change in angular velocity over three successive time points was determined.

The angular acceleration value for each animal is reported to one significant digit to account for digital filtering. Best fit curves were generated using non-linear regression. For linear velocity and linear acceleration, the acceleration and deceleration phases were fit separately.

### Behavior

Functional and neurobehavioral testing except for latency to righting reflex (LRR), Rotarod (RR), and neuroscore (NS) was carried out in a dedicated behavior room during workday hours. This room was equipped with light, sound, and humidity controls and isolated from external noise. Mice were allowed to acclimate in this room for at least 1 hour prior to testing. Illuminance was measured and set independently at the start of each test (40 lux for Morris water maze testing; 20 lux for all other tests) using a lux meter (Sper Scientific 840006, Scottsdale, AZ, USA) at the level of the test apparatus. A white noise machine (Marpac Dohm-DS, Wilmington, NC, USA) was set to deliver 60 dB at the test apparatus, measured with a sound level meter (Lafayette Instrument SL-A, Lafayette, IN, USA). LRR, RR, and neuroscore were performed in separate environmentally-controlled facilities during workday hours. To eliminate scents all testing surfaces were cleaned with 70% ethanol prior to testing and between animals. For Morris water maze, elevated plus maze, social interaction, and open field testing an overhead camera recorded all mouse paths, which were subsequently analyzed using SMART. Heat maps of dwell time were prepared by exporting coordinate data from SMART, processing these coordinates into individual heat maps using a custom MATLAB script (Mathworks, Natick, MA, USA), overlaying individual heat maps by group using Photoshop, and then transforming to a color look-up table in ImageJ/FIJI (NIH, Bethesda, MD, USA). LRR, neuroscore, and tail suspension were analyzed manually by blinded observers. All other behavioral analyses were performed using automated animal tracking with SMART or device-integrated monitoring (RR). Experimenters were blind to treatment group during testing. See Supplementary Figure 1 for the time line of behavioral testing.

### Latency to Righting Reflex (LRR) and nestlet testing

Following injury animals were observed in a quiet, temperature-controlled environment for righting reflex by placing them on their side and measuring the length of time from the moment of impact until the animals rolled onto their abdomens. Nestlet testing was performed as previously described^57^ by singly housing animals with a pre-weighed cotton nestlet (average weight 2.87 ± 0.04 g) and re-weighing the nestlet at post-injury intervals to determine the amount of undisturbed material.

### Open field test

Mice were placed in the corner of a 44.5 × 44.5 cm opaque box and allowed to explore for 5 minutes. The amount of time spent moving was calculated using SMART. To monitor thigmotaxic behavior, average distance from the wall of the open field box was calculated by defining square concentric zones spaced 1.5 cm apart (15 in total) using SMART and deriving a time-weighted average.

### Neuroscore (NS) testing

Neurobehavioral testing was performed during the first week post-injury. Animals underwent a battery of tests that assessed general activity, fore/hind-paw function, grip strength, and balance, and were scored based on performance on a standardized rating scale (see Supplementary Information).

### Rotarod (RR)

Testing was performed on a Rotamex rotarod device (Columbus Instruments; Columbus, OH, USA). Animals were trained on three consecutive days prior to injury to walk on a 3 cm rotating rod. On the first day of training animals were acclimated to standing on an idle rod for one minute followed by two, one-minute sessions at a constant speed of 2 RPM. Following initial acclimation and on the following two days animals underwent 3 trials with the rod accelerating from 2–20 RPM at 0.1 RPM/sec. During training if an animal fell from the rod it was immediately replaced until the max speed was reached. Following injury animals were tested in 3 sessions with the rod accelerating identically to training trials. The latency to fall was recorded and averaged.

### Elevated Plus Maze

The elevated plus maze test was performed using a custom maze elevated 50 cm above board with arms that were 30 cm long and 5 cm wide. Closed arms were enclosed by walls 16 cm high. Open arms had a 2 mm rail to prevent falls. Mice were placed at the end of a closed arm and allowed to explore the maze for 5 min. A 14 × 14 cm central zone was defined such that animals outside of this zone on the open arm had all 4 paws on the open arm. Open arm, central area, and closed arm time were quantified in SMART.

### Morris Water Maze (MWM)

A 120 cm diameter pool made opaque by the addition of white tempera paint was used for all trials. Mice underwent four trials each day. They were inserted into the pool at a different location for each trial. The order of the insertion points was changed daily. For visible platform testing, a visible flag was placed on the platform. Following visible testing mice underwent 4 days of hidden platform training with the escape platform relocated to a new quadrant of the pool and lowered 1 cm below the surface. An opaque, featureless curtain was closed around the pool for these trials. Prominent visual cues were hung at intervals on this curtain. The day following hidden platform testing mice underwent a single probe trail in which the platform was removed and mice were inserted at a consistent, distant point from the prior platform location. The average distance from the prior platform location over 15 seconds was measured in SMART.

### Social Interaction and social novelty

Test mice were singly-housed for 24 hours prior to testing for Crawley’s sociability and preference for social novelty in a 42 × 70 cm, 3-chamber apparatus as previously described^58^. Two types of mice were used for testing: test mice (injured or control) and stimulus mice. Stimulus animals were of the same strain and sex as the subject mice, but were older. Initially the test mouse was placed in the box and allowed to habituate to the entire apparatus for 5 minutes. For the social interaction test, the mouse was then confined in the middle chamber while a stimulus mouse was placed into one of the wire cages and a dummy mouse was placed into the other. The test mouse was then allowed to explore for ten minutes and the amount of time interacting, defined as the time the test mouse’s head was within 1.5 cm of the stimulus/dummy mouse cage, was quantified using SMART. A sociability index was calculated by determining the ratio of time with stimulus to time with dummy mouse, and then normalizing to control mice for each cohort.

Following social interaction testing the test mouse was again confined to the middle chamber and the dummy mouse was replaced with a novel stimulus mouse for social novelty testing. Again the test mouse was allowed to explore for 10 minutes and an analogous social novelty index was calculated. Stimulus mice were rotated between test mice such that >30 min of rest was provided between re-exposure to a new test mouse. Following social novelty testing, test mouse olfactory function was assessed by placing a flavored cereal piece (Chocolate Toasted O’s) beneath bedding in a corner of a clean cage. The amount of time it took the animal find the cereal was recorded manually.

### Tail Suspension

The tail suspension test was performed as previously described^59,60^. Briefly, mice were suspended by the tail with adhesive tape from a rod at a height of 30 cm. The tail was passed through a cardstock paper cone (5.4 cm at the base, 5.5 cm tall, replaced for each animal) to prevent climbing to the rod during testing. The test was recorded for 6 minutes. Time immobile, defined as lack of movement except for that due to momentum from prior movement^59^, was quantified by a blinded observer.

### Tissue collection

At the appropriate time post-injury mice were administered a lethal dose of isoflurane and transcardially perfused with 4 °C 0.1 M phosphate buffered saline (PBS). Following clearing of blood, 4% paraformaldehyde in PBS (PFA) at 4 °C was perfused for 6 minutes. Brains were then extracted and post-fixed in PFA overnight at 4 °C followed by equilibration in 30% sucrose in PBS for at least 24 hrs. Brains were sectioned on a freezing microtome (Microm HM 430, ThermoFisher Scientific, Waltham, MA, USA) at 50 µm.

### Immunhistochemistry

The following primary antibodies were used: Iba-1 (DAKO, Santa Clara, CA, USA, cat. #019-19741, 1:1000), β-APP (Life Technologies, Carlsbad, CA, USA, cat. #512700, 1:1000), biotinylated anti-mouse IgG (Vector Labs, Burlingame, CA, USA, cat. # BA-9200, 1:1000), SMI-31 (Calbiochem, Billerica, MA, USA, cat. #NE1022, 1:1000). Sections were rinsed in Tris-buffered saline (TBS), treated with 6% hydrogen peroxide in 50:50 TBS:methanol to block endogenous peroxidases, blocked with 3% normal goat serum (Vector Labs, Burlingame, CA, USA, cat. #S-1000) with 0.25% Triton X-100 (Sigma-Aldrich, St. Louis, MO, USA, cat. #T8787), and then incubated with primary antibodies overnight at 4 °C with gentle shaking. The following day sections were treated with biotinylated goat anti-rabbit antibody (Vector Labs, Burlingame, CA, USA, cat. #BA-1000), except for IgG staining, and then with ABC reagent (Vector Labs, Burlingame, CA, USA, cat. #PK-6100) using a mixture of 1:400 solution A:B in TBS for 1 hour. Sections were developed using a 0.25 mg DAB/1 ml TBS solution with nickel chloride intensification. For SMI-31 staining, the primary antibody was mixed 1:1 with biotinylated anti-mouse FAb fragments (Jackson ImmunoResearch, West Grove, PA, USA) at room temperature for 1 hour followed by incubation with an excess of normal mouse serum (Life Technologies, Carlsbad, CA, USA, cat. #10410) prior to tissue application. Development with ABC and DAB then proceeded as above.

### Histochemical stains

Cresyl violet staining (FD Neuro, Columbia, MD, USA, cat. #PS102-1) was performed by first immersing the tissue in dye for 20 minutes, then rinsing with water and differentiating in ethanol. Perl’s staining of iron was performed using the Prussian blue stain (Polysciences, Warrington, PA, USA, cat. #24199-1) per manufacturer’s instructions followed by DAB intensification as previously described^61^. Fluoro-jade C (Histo-Chem Inc., Jefferson, AR, USA, cat. #1FJC) staining was performed on charged slides following manufacturer’s instructions with the modification that the fluoro-jade C incubation was performed for 1 hour at 4 °C.

### Histological analysis

Tissue sections were scanned on a Zeiss Axioscan (Carl Zeiss AG, Oberkochen, Germany) using the following objectives: 5× for CV, β-APP, SMI-3, Iba-1; 10× for Perl’s. Images were exported with Zeiss Zen software and processed for quantification. Fluoro-jade C+ cells were counted manually in all hippocampal sub-regions on a Nikon Eclipse 80i epifluorescent microscope (Nikon Corporation, Tokyo, Japan) at 20× magnification. Bilateral hippocampi were analyzed from 3 sections per animal between Bregma −1.55 and −2.155 mm (Allen Brain Atlas, Allen Institute, Seattle, Washington, USA). For analysis of atrophy at 30 days post-injury (dpi), semi-automated thresholding was applied with ImageJ/FIJI to cresyl violet-stained sections to isolate brain tissue. Partial brain volume was calculated using the Cavalieri method^62^ over 10 contiguous sections per animal spaced at 300 µm covering 3 mm in the rostral-caudal axis stretching from Bregma +1.355 to −2.355 (Allen Brain Atlas, Allen Institute, Seattle, Washington, USA). For quantification of β-APP, the Find Maxima command in ImageJ/FIJI was used to identify axonal swellings (noise tolerance 20). This technique was validated against stereological analysis (Pearson r = 0.96, Supplementary Fig. 2). For analysis of iron accumulation, a fixed threshold of 0–50 was first applied to grayscale images in ImageJ/FIJI. The thresholded volume of Perl’s-stained tissue was then measured using the Cavalieri method^62^. Microglial density was determined in ImageJ/FIJI using the Analyze Particles command on grayscale images with a size cut-off set to 10–100 μm^2^ following isolation of microglia using the Autolocal Threshold command (Sauvola filter). All histological analyses were performed blinded.

### Diffusion Tensor Imaging

Mouse brains were collected 2 days after sham or 1.7 J modCHIMERA injury per histological methods through post-fixation, equilibrated in PBS for 48 hours, and imaged *ex vivo* with a 4.7 T Agilent DirectDrive^TM^, actively-shielded small animal MRI system (bore 40 cm long, 10 cm inner diameter) with gradient coils capable of producing linear magnetic field gradients up to 60 G/cm with a 200 μs rise time. A custom-made cylinder-type radio frequency coil with a 2.4 cm inner diameter was used for both transmission and reception. The main axis of the brain was positioned parallel to that of the coil. Diffusion weighted images were collected with a diffusion spin-echo imaging sequence^63^ with the following acquisition parameters: repetition time 1.5 sec, spin echo time 36 ms, time between application of gradient pulses 20 ms, diffusion gradient duration 7 ms, field of view 15 mm × 15 mm, and data matrix 128 × 128 yielding an in-plane resolution of 117 × 117 μm with a slice thickness of 0.5 mm. Images were obtained with diffusion sensitizing gradients applied in 15 orientations (ICOSA 15)^64^. Two b values were used: 0 and 2000 s/mm^2^. 12 consecutive coronal slices were collected covering midbrain to anterior commissure. Fractional anisotropy (FA) was calculated using robust estimation of tensors by outlier rejection (RESTORE)^65^ in tolerably obsessive registration and tensor optimization indolent software (TORTOISE, https://science.nichd.nih.gov/confluence/display/nihpd/TORTOISE). Hand-drawn, multislice ROIs were defined by pre-specified anatomical boundaries on FA and b0 images. A voxel-weighted FA measure was calculated for each ROI.

### Statistical analysis

All statistical analyses were performed with Prism v7.0 (Graph Pad, La Jolla, CA, USA). All data sets were first tested for normality using the Shapiro-Wilk normality test. Any non-normally distributed data set that could be normally transformed was. β-APP in corpus callosum/fimbria/hippocampal commissure, social interaction, tail suspension, elevated plus maze, and iron accumulation, and MWM probe trial results were tested by one-way ANOVA followed by the Holm-Bonferroni post-hoc test. The Kruskal-Wallis test followed by Dunn’s post-test was used for RR, open field test, neuroscore, MWM visible platform testing, fluoro-jade C, brain volume, brain weight, and β-APP in anterior commissure. MWM hidden platform testing and Thigmotaxis were analyzed with a two-way repeated measures ANOVA followed by Holm-Bonferroni post-hoc test. Diffusion tensor imaging was analyzed by Mann-Whitney U test with correction for multiple comparison for each region. Dichotomous distracting injuries (*e.g*., skull fracture) were analyzed with the Fisher’s exact test. All tests were 2-tailed. Statistical significance was set at *p* < 0.05. For all figures, *p < 0.05, **p < 0.01, **p < 0.001. All data are represented as mean ± SEM.

### Data availability

The datasets generated during and/or analyzed during the current study are available from the corresponding author on reasonable request.

## Electronic supplementary material

Supplementary Movie 1

Supplementary Movie 2

Supplemental information
