# Supplementary material for: modCHIMERA: a novel murine closed-head model of moderate traumatic brain injury
Source: Sci Rep. 2018 May 16;8:7677. doi: 10.1038/s41598-018-25737-6 (PMC5955903; doi:10.1038/s41598-018-25737-6)
Supplement: Supplementary file 3 — Supplemental information [file 41598_2018_25737_MOESM3_ESM.pdf]

TITLE:

modCHIMERA: a novel murine closed-head injury model of moderate traumatic brain injury

AUTHORS:

Sauerbeck AD, Fanizzi C, Kim JH, Gangolli M, Bayly PV, Wellington CL, Brody DL, Kummer TT*

Andrew D Sauerbeck, PhD
Postdoctoral Researcher
Washington University
School of Medicine
Department of Neurology
Saint Louis, MO, USA
660 S. Euclid
Campus Box 8111
St. Louis, MO 63100
Phone: 314-362-1378
Fax: 314-362-3279
Email:
Andrew.sauerbeck@wustl.edu

Claudia Fanizzi, MD
Visiting Scientist
Washington University
School of Medicine
Department of Neurology
Saint Louis, MO, USA
660 S. Euclid
Campus Box 8111
St. Louis, MO 63100
Phone: 314-362-1378
Fax: 314-362-3279
Email:
fanizzi.claudia@gmail.com

Joong H Kim, PhD
Instructor
Washington University
School of Medicine
Department of Neurology
Saint Louis, MO, USA
660 S. Euclid
Campus Box 8111
St. Louis, MO 63100
Phone: 314-362-1378
Fax: 314-362-3279
Email: jkim35@wustl.edu

Mihika Gangolli, BS
Graduate Student
Washington University
School of Medicine
Department of Biomedical
Engineering
Saint Louis, MO, USA
660 S. Euclid
Campus Box 8111
St. Louis, MO 63100
Phone: 314-362-1378
Fax: 314-362-3279
Email:
mihika.gangolli@wustl.edu

Philip V Bayly, PhD
Professor
Department Chair
Washington University
Mechanical Engineering and
Materials Science
Campus Box 1185
Urbauer Hall, Room 319E
Phone: 314-935-6081
Email: pvb@wustl.edu

Cheryl L Wellington, PhD
Professor
University of British Columbia
Department of Pathology and
Laboratory Medicine
2215 Wesbrook Mall,
Vancouver, BC V6T 1Z3
Phone: 604-827-3769
Fax: 604-822-0361
Email: wcheryl@mail.ubc.ca

David L Brody, MD, PhD
Professor
Washington University
School of Medicine
Department of Neurology
660 S. Euclid
Campus Box 8111
St. Louis, MO 63100
Phone: 314-362-1378
Fax: 314-362-3279
Email: brodyd@wustl.edu

*Corresponding Author:

Terrance T Kummer, MD, PhD
Assistant Professor
Washington University
School of Medicine
Department of Neurology
660 S. Euclid
Campus Box 8111
St. Louis, MO 63100
Phone: 314-362-1378
Fax: 314-362-3279
Email: kummert@wustl.edu

SUPPLEMENTARY INFORMATION

Sauerbeck *et al*., 2018

1. Supplementary Figures 1-9
2. Supplementary Tables 1 and 2
3. Neuroscore Protocol
4. Supplementary Movies 1 and 2 (captions)

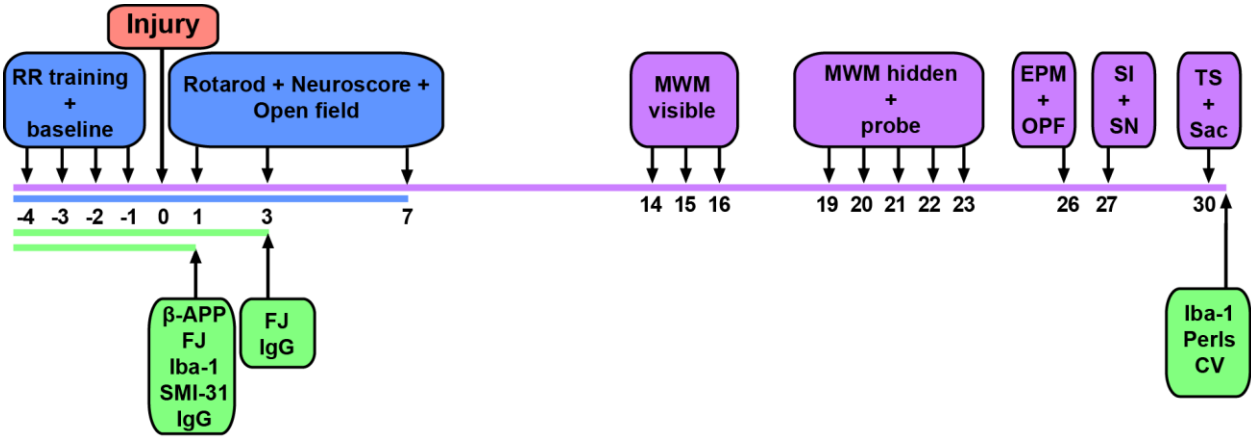

Supplementary Figure 1: Experimental timeline

Colors represent acute behavioral tests (blue), modCHIMERA injury (red), subacute/chronic behavioral tests (purple), and histological endpoints (green). Horizontal lines indicate individual cohorts of animals.

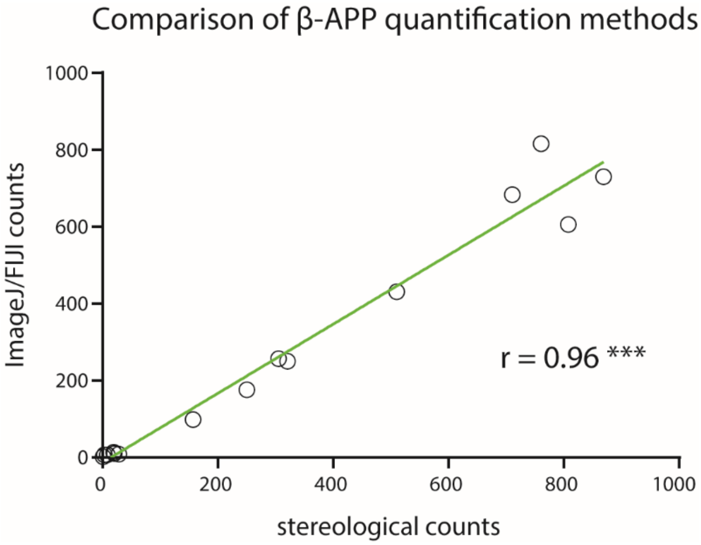

Supplementary Figure 2: Validation of β-APP quantification vs. stereological counting

β-APP puncta in the anterior commissure across experimental groups were quantified using both the ImageJ/FIJI method (see Methods) and using Stereo Investigator with a grid size of 100 x 100 μm and a counting frame of 100 x 100 μm (entire ROI quantified).

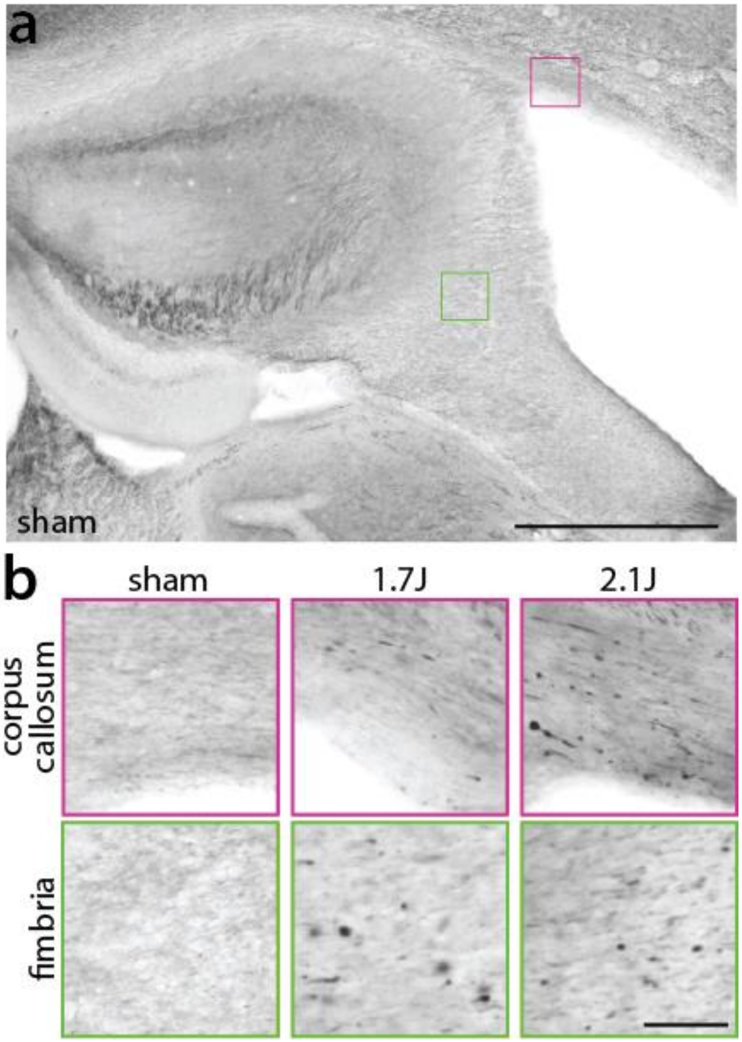

Supplementary Figure 3: SMI-31 staining of white matter pathology. Control animals do not exhibit SMI-31-positive puncta (a, b). Following modCHIMERA, however, SMI-31-positive puncta indicative of axonal injury are evident in the corpus callosum and fimbria of animals injured at both intensities (b). Similar puncta are observed in anterior commissure and hippocampal commissure (data not shown). Scale bar (a) 500 μm; (b) 50 μm.

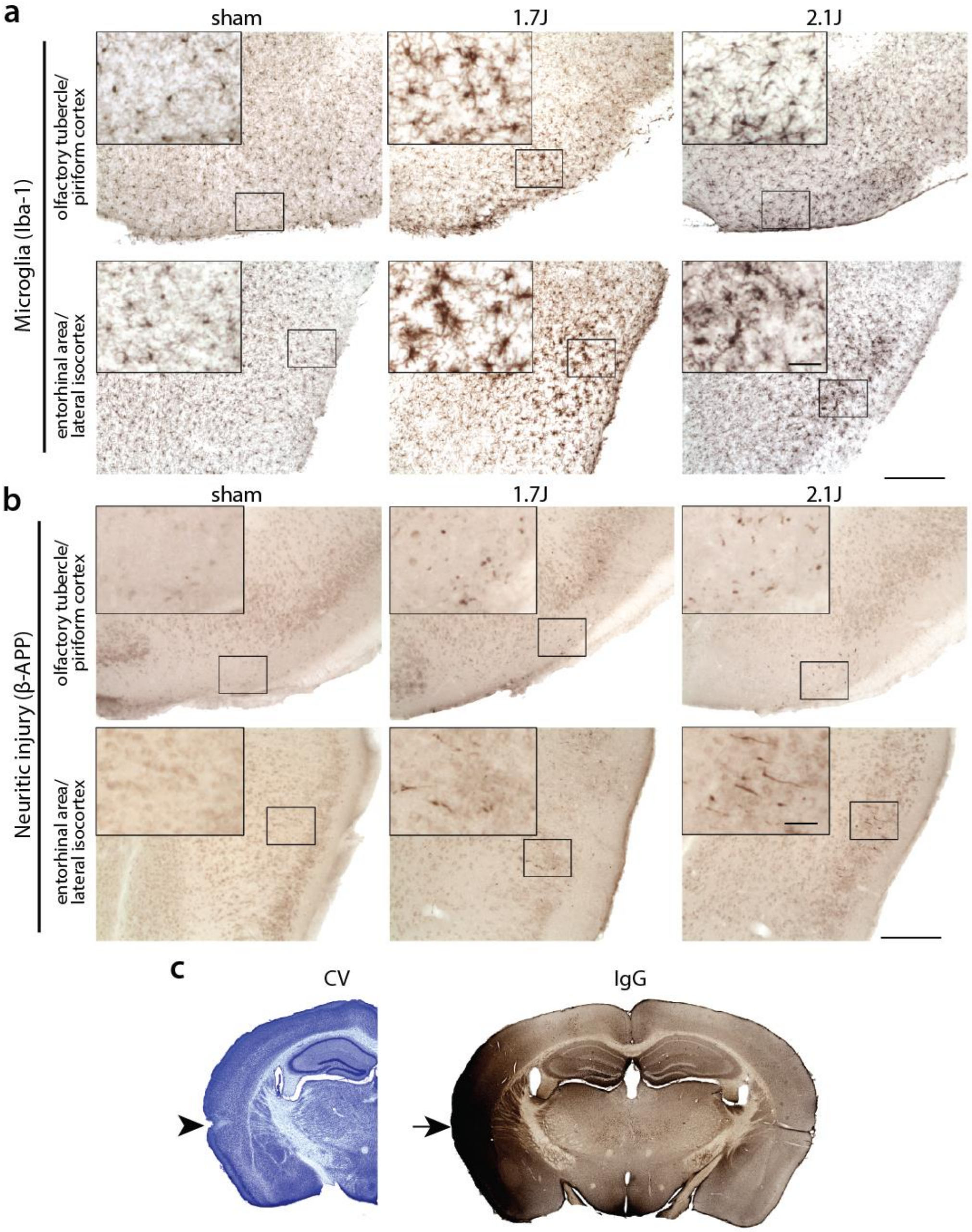

Supplementary Figure 4: Superficial cortical injury in modCHIMERA. Microglial activation in superficial cortical layers of anteroinferior and posterolateral cortex detected by Iba-1 immunoreactivity after modCHIMERA at 1 dpi (a). Boxed region shown at higher magnification in inset. Note increase in number and size of microglia after injury in these regions. β-APP positive puncta indicative of neuritic injury in the same regions as above at 1 dpi (b). Boxed region shown at higher magnification in inset. Cresyl violet staining at one-month post-injury demonstrating a superficial cortical injury in lateral isocortex (arrowhead), with surrounding halo of IgG labeling (c; arrow). Scale bars low magnification 250 μm; high magnification 50 μm.

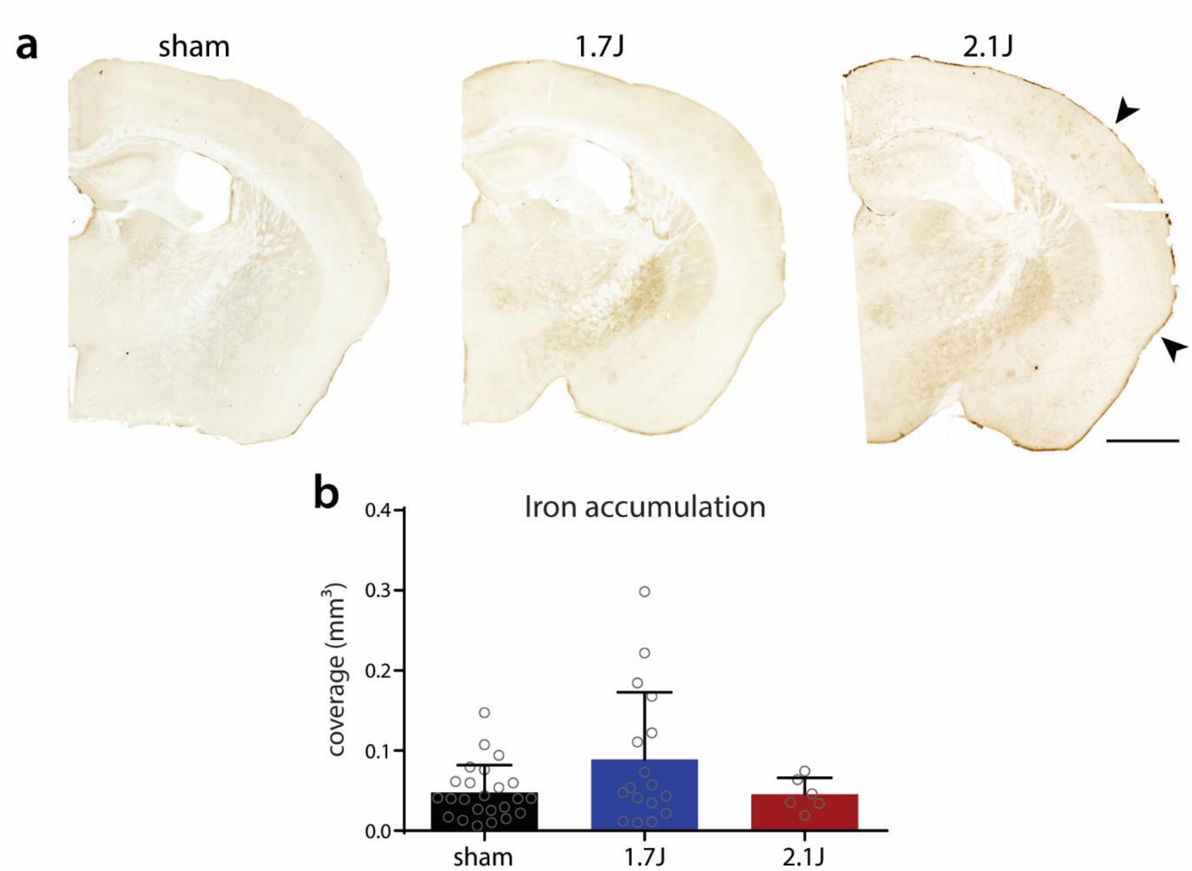

Supplementary Figure 5: Vascular integrity after modCHIMERA. . Perl's staining for iron deposition at 30 dpi (a). Note lack of evidence of parenchymal bleeding following modCHIMERA. Arrowheads indicate meningeal iron accumulation following highest intensity injury (see Results). Quantification of brain iron deposition (b). Data represented as mean ± SEM. Iron accumulation tested by one-way ANOVA followed by Holm-Bonferroni post-hoc test. Scale bar (a) and (d) 1 mm. n = 6-24/group.

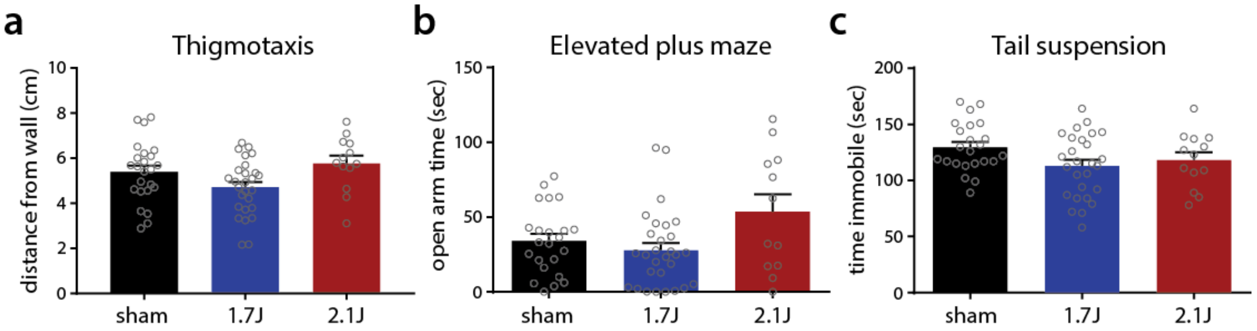

Supplementary Figure 6: Anxiety- and depressive-like behavior after modCHIMERA. Mean distance from the wall of the testing chamber as a measure of thigmotaxic exploration at 26 dpi (a). Amount of time spent on open arms of the elevated plus maze at 26 dpi (b). Note neither thigmotaxic behavior nor time on open arm reveal differences in anxiety-related behaviors between control and injury groups. Amount of time immobile during the tail suspension test at 30 dpi does not reveal differences in depressive-like behavior between groups (c). Data represented as mean ± SEM. Thigmotaxis, elevated plus maze, and tail suspension analyzed with one-way ANOVA followed by Holm-Bonferroni post-hoc test. n = 6-33/group.

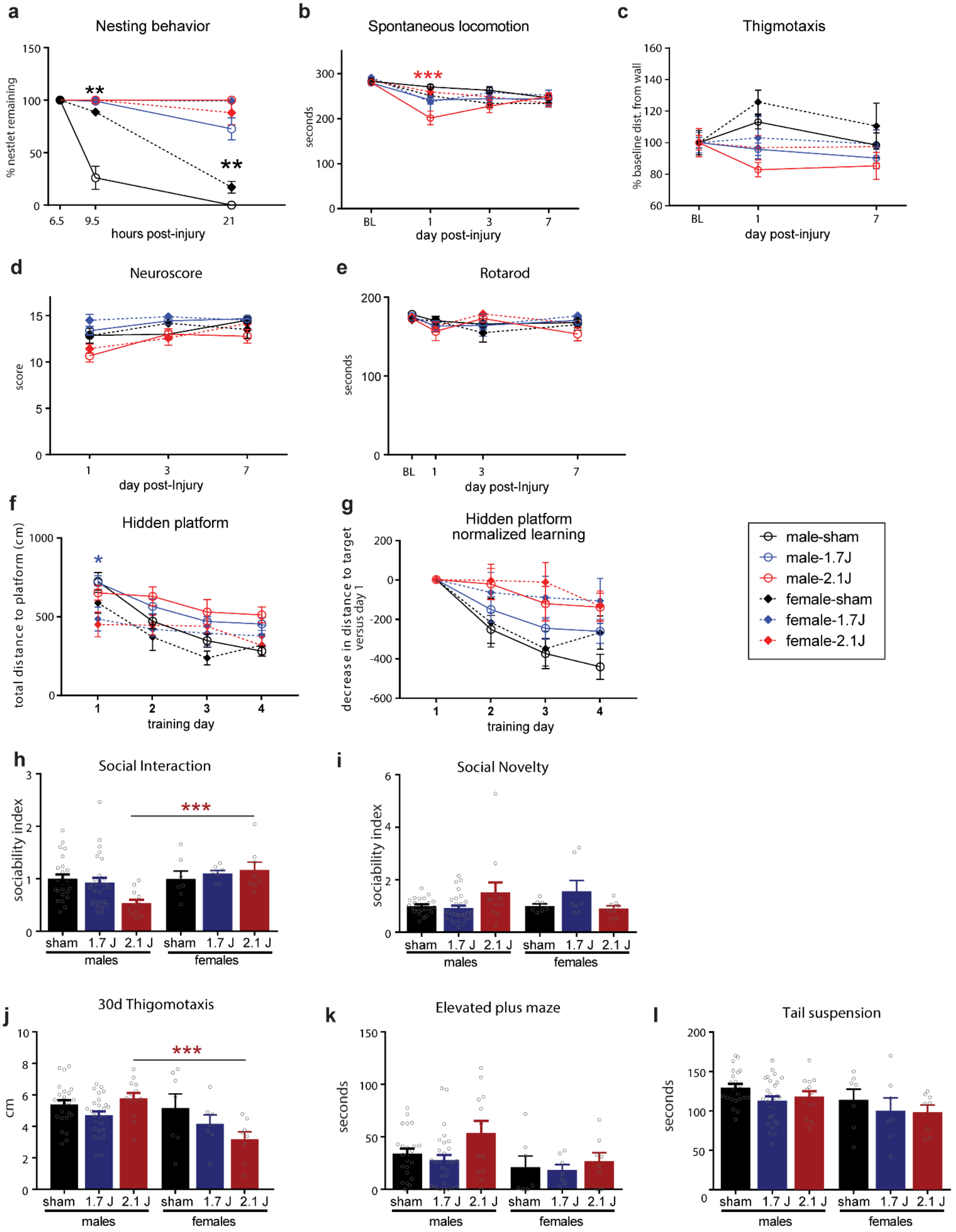

Supplementary Figure 7. Assessment of behavioral differences in male and female mice after modCHIMERA. Uninjured female mice exhibit reduced nest building behavior compared to male mice,

while nest building between male and female injured animals at either 1.7 J or 2.1 J was not significantly different (a; male mice sham n=6, 1.7 J n=10, 2.1 J n=3; female mice sham n=4, 1.7 J n=5, 2.1 J n=5). Female mice injured at 2.1 J only were more active than male mice in the open field test at 1 day post-injury, but were otherwise statistically indistinguishable (b; male mice sham n=24, 1.7 J n=12, 2.1 J n=24; female mice sham n=7, 1.7 J n=7, 2.1 J n=8). No statistical differences were observed in acute thigmotaxic behavior between male and female mice, though female mice did tend to explore further from the wall during the first week post-injury than male mice in all groups including controls (c; male mice sham n=24, 1.7 J n=12, 2.1 J n=24; female mice sham n=7, 1.7 J n=7, 2.1 J n=8). Female mice exhibited similar performance to male mice on the neuroscore test (d; male mice 1 dpi sham n=8, 1.7 J n=9, 2.1 J n=11; 3 dpi sham n=8, 1.7 J n=9, 2.1 J n=7; 7 dpi sham n=8, 1.7 J n=9, 2.1 J n=3; female mice 1-7 dpi sham n=6, 1.7 J n=8, 2.1 J n=7) and rotarod (e; male mice 1 dpi sham n=8, 1.7 J n=8, 2.1 J n=10; 3 dpi sham n=8, 1.7 J n=8, 2.1J n=7; 7 dpi sham n=8, 1.7 J n=8, 2.1 J n=3; female mice 1-7 dpi sham n=6, 1.7 J n=8, 2.1 J n=7e) in all groups. On the first day of Morris water maze hidden platform training, female mice injured at 1.7 J took a shorter path to the hidden platform location than male mice (f; male mice sham n=24, 1.7 J n=28, 2.1 J n=12; female mice sham n=7, 1.7 J n=7, 2.1 J n=8). Interestingly, all females exhibited a trend to shorter path lengths vs. male mice. This reached statistical significance for animals injured at 1.7 J on day 1 only. Normalized analysis of learning revealed that male and female mice exhibit a similar magnitude of learning when assessed by reduction in path distance to the hidden platform over the four days of training (g; male mice sham n=24, 1.7 J n=28, 2.1 J n=12; female mice sham n=7, 1.7 J n=7, 2.1 J n=8). Female mice injured at 2.1 J exhibited a reduction in social interaction deficits compared to male mice (h; male mice sham n=24, 1.7 J n=28, 2.1 J n=12; female mice sham n=7, 1.7 J n=7, 2.1 J n=8). No significant differences were observed in social novelty testing between male and female mice (i; male mice Sham n=24, 1.7 J n=28, 2.1 J n=12; female mice sham n=7, 1.7 J n=7, 2.1 J n=8). Female mice injured at 2.1 J remained closer to the wall during open field testing at 30 days post-injury compared to male mice, indicative of prolonged anxiety-related behavior in females vs. males at this energy level (j; male mice sham n=24, 1.7 J n=28, 2.1 J n=13; female mice sham n=7, 1.7 J n=7, 2.1 J n=8). No statistically significant differences were observed between male and female animals in elevated plus maze (k; male mice sham n=24, 1.7 J n=29, 2.1 J n=12; female mice sham n=7, 1.7 J n=7, 2.1 J n=8) or tail suspension (l; male mice sham n=23, 1.7 J n=27, 2.1 J n=13; female mice sham n=7, 1.7 J n=7, 2.1 J n=8) tests. Data represented as mean ± SEM. Statistical analysis of spontaneous activity, acute thigmotaxis, neuroscore, rotarod, and morris water maze was performed using a two-way ANOVA between males and females within each injury condition. Statistical analysis of nesting behavior, social interaction, chronic thigmotaxis, elevated plus maze, and tail suspension was performed with a t-test between males and females within each injury condition. Asterisk color indicates group wise comparison reaching statistical significance (black = difference between shams, blue = difference between 1.7 J animals, red = difference between 2.1 J animals).

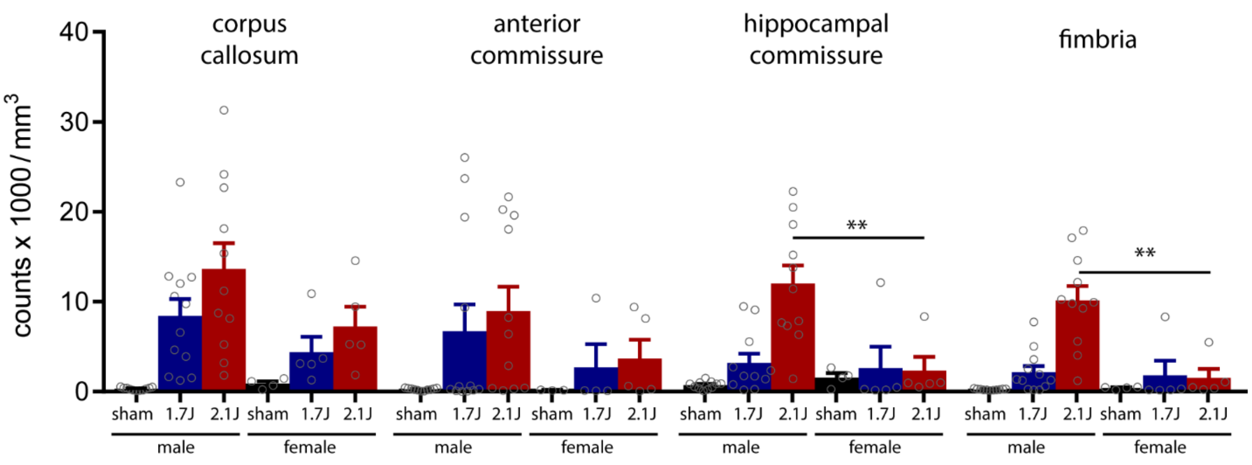

Supplementary Figure 8. Assessment of white matter injury in male and female mice after modCHIMERA. White matter injury, detected by the accumulation of β-APP, was not statistically different between male and female mice injured at either 1.7 J or 2.1 J in the corpus callosum or anterior commissure. White matter injury was significantly reduced in the hippocampal commissure and fimbria in female vs. male mice injured at 2.1 J only. Data represented as mean ± SEM. Statistical analysis was performed with a t-test between male and female mice within each injury condition. N = male mice sham n=11, 1.7 J n=12, 2.1 J n=11; female mice sham n=4, 1.7 J n=5, 2.1 J n=5.

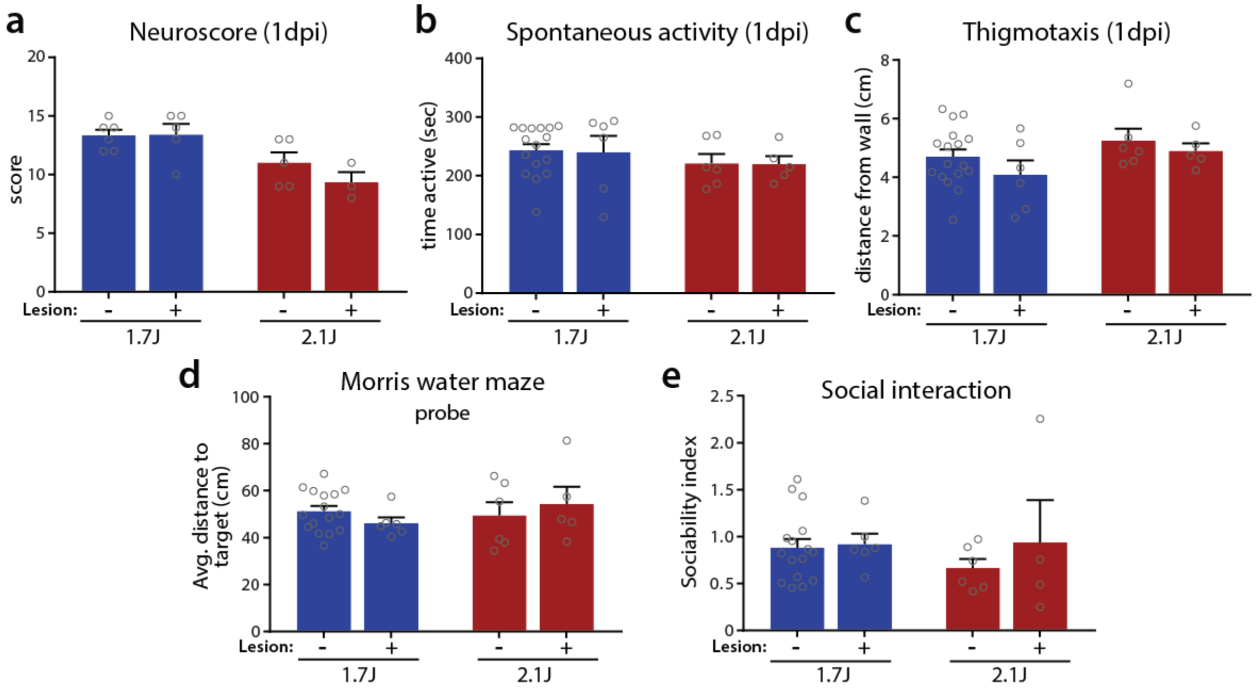

Supplementary Figure 9: Effect of macroscopic focal injury on behavioral outcomes after modCHIMERA: Comparison of 1 dpi neuroscore (a), 1 dpi spontaneous activity in the open field test (b), 1 dpi thigmotaxis (c), Morris water maze probe test (d), and social interaction (e) between injured animals with and without macroscopic focal cerebral lesions. Data represented as mean ± SEM. Comparisons between animals with and without focal lesions were performed within each injury severity with a Mann Whitney U test. N = 3-17/group.

	1.7J	2.1J	
n	113	111	*p*
inclusion (%)	84.1	58.5	<0.0001
death (%)	8.8	23.4	0.0034
skull fracture (%)	1.8	18	<0.0001
subdural hemorrhage (%)	4.4	4.5	>0.9999

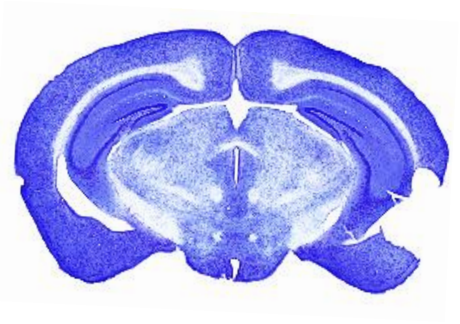

Supplementary Table 1: Morbidity and mortality with modCHIMERA. Inclusion and death rates for each injury severity, along with most common distracting injuries are presented. Data analyzed with the Fischer-exact test. Image depicts an example of an exclusionary lesion detected in the posterolateral cortex from a cortical contusion/laceration associated with skull fracture.

	mouse equivalent*			modCHIMERA	
	threshold	average	severe	1.7 J	2.1 J
**Linear velocity (m/s)**	5.4	7.2	9.2	8.72	9.2
**Linear acceleration (g)**	967.4	1349.64	1731.9	1398	1446
**Angular velocity (rad/sec)**	270.5	480.2	690	195	230
**Angular acceleration (krad/sec^2)**	879.6	1224.9	1570.2	90	90

***mouse equivalent values scaled from NFL values [1] with mouse scaling factor [2].**

Supplementary Table 2: Comparison of human-equivalent brain injury biomechanics with modCHIMERA. Human-equivalent injury levels were calculated using data from NFL concussion published in Viano et al. 2009 [1] using a scaling factor of 13.8 per Namjoshi et al. 2014 [2]. Human kinematic measurements were scaled using the equal-stress equal-velocity approach [1, 2]. Per this biomechanical analysis, linear velocity between humans and rodents is not scaled. Linear acceleration and angular velocity are scaled using a scaling factor 13.8 to account for size differences between the human and mouse brain. Rotational acceleration is scaled using the square of this scaling factor. modCHIMERA exceeds human-equivalent brain injury thresholds for linear velocity and linear acceleration of the head, but not for angular velocity or angular acceleration.

[1] Viano, D.C., Hamberger, A., Bolouri, H. & Saljo, A. Concussion in professional football: animal model of brain injury--part 15. Neurosurgery 64, 1162-1173 (2009).

[2] Namjoshi, D.R., et al. Merging pathology with biomechanics using CHIMERA (Closed-Head Impact Model of Engineered Rotational Acceleration): a novel, surgery-free model of traumatic brain injury. Mol Neurodegener 9, 55 (2014).

Neuroscore testing

Test	Scoring
**Spontaneous Activity:** Place mouse in empty, clean cage without top and observe from a distance for 1 min	**0:** No movement or minimal movement<br>**1:** Animal approaches < 3 walls<br>**2:** Animal approaches 3 or more walls but does not reach for rim of cage<br>**3:** Normal exploration. Reaches for upper rim of cage and approaches at least three walls.
**Symmetry of Movement:** Hold animal by the tail in the air near floor of cage and observe limb movement while reaching for floor, and then allow to explore cage while watching rear limb movements for symmetry for 30 seconds	**0:** One or both forelimbs paralyzed or move minimally<br>**1:** One or both forelimbs impaired<br>**2:** Normal reaching, abnormal walking/running<br>**3:** All extremities extend symmetrically and walking is normal
**Climbing:** Place animal on mesh and flip upside down**,** hold upside down for 30 seconds	**0:** Unable to hang from mesh<br>**1:** Able to hang on mesh briefly but falls before trial complete<br>**2:** Able to hang but unable to displace on mesh (move all 4 limbs)<br>**3:** Able to hang and displace on mesh
**Balance & Coordination:** Place animal on 1 cm diameter pole, and turn pole so that mouse is at the bottom, give 30 seconds to complete task	**0:** Unable to hold the pole<br>**1:** Able to hold for a few seconds but falls or cannot right<br>**2:** Able to hold on pole and get to the top surface but cannot walk<br>**3:** Able to hold on pole and get to the top surface and walk
**Beam walk 1**: Place animal on the end of 8mm x 8mm square beam. Animal must walk 30cm in 1 minute	**0:** Does not walk 30cm<br>**1:** Walks 30cm
**Beam walk 2**: Place animal on the end of 5mm x 5mm square beam. Animal must walk 30cm in 1 minute	**0:** Does not walk 30cm<br>**1:** Walks 30cm
**Beam walk 3**: Place animal on the end of 8mm diameter round beam. Animal must walk 30cm in 1 minute	**0:** Does not walk 30cm<br>**1:** Walks 30cm
	Total possible score: 15

Supplementary Movie 1: Video showing impact and full rotation of animal.

Supplementary Movie 2: High-speed video of initial impact and head movement.
